# Scientific concepts and methods for moving persistence assessments into the 21st century

**DOI:** 10.1002/ieam.4575

**Published:** 2022-02-23

**Authors:** Russell Davenport, Pippa Curtis‐Jackson, Philipp Dalkmann, Jordan Davies, Kathrin Fenner, Laurence Hand, Kathleen McDonough, Amelie Ott, Jose Julio Ortega‐Calvo, John R. Parsons, Andreas Schäffer, Cyril Sweetlove, Stefan Trapp, Neil Wang, Aaron Redman

**Affiliations:** ^1^ School of Engineering Newcastle University Newcastle upon Tyne UK; ^2^ Environment Agency Wallingford Oxfordshire UK; ^3^ Bayer AG, Crop Science Division, Environmental Safety Monheim Germany; ^4^ LyondellBasell Rotterdam The Netherlands; ^5^ Eawag, Swiss Federal Institute of Aquatic Science and Technology Dübendorf Switzerland; ^6^ Department of Chemistry University of Zürich Zürich Switzerland; ^7^ Syngenta, Product Safety, Jealott's Hill International Research Centre Bracknell UK; ^8^ P&G, Global Product Stewardship Mason Ohio USA; ^9^ European Centre for Ecotoxicology and Toxicology of Chemicals (ECETOC) Brussels Belgium; ^10^ Instituto de Recursos Naturales y Agrobiología de Sevilla Consejo Superior de Investigaciones Científicas Sevilla Spain; ^11^ Institute for Biodiversity and Ecosystem Dynamics University of Amsterdam Amsterdam The Netherlands; ^12^ RWTH Aachen University, Institute for Environmental Research Aachen Germany; ^13^ L'Oréal Research & Innovation Environmental Research Department Aulnay‐sous‐Bois France; ^14^ Department of Environmental Engineering Technical University of Denmark Bygningstorvet Lyngby Denmark; ^15^ Total Marketing & Services Paris la Défense France; ^16^ ExxonMobil Petroleum and Chemical Machelen Belgium

**Keywords:** Biodegradation, Persistence assessment, Biodegradability, Bioavailability, Degradation half‐lives

## Abstract

The evaluation of a chemical substance's persistence is key to understanding its environmental fate, exposure concentration, and, ultimately, environmental risk. Traditional biodegradation test methods were developed many years ago for soluble, nonvolatile, single‐constituent test substances, which do not represent the wide range of manufactured chemical substances. In addition, the Organisation for Economic Co‐operation and Development (OECD) screening and simulation test methods do not fully reflect the environmental conditions into which substances are released and, therefore, estimates of chemical degradation half‐lives can be very uncertain and may misrepresent real environmental processes. In this paper, we address the challenges and limitations facing current test methods and the scientific advances that are helping to both understand and provide solutions to them. Some of these advancements include the following: (1) robust methods that provide a deeper understanding of microbial composition, diversity, and abundance to ensure consistency and/or interpret variability between tests; (2) benchmarking tools and reference substances that aid in persistence evaluations through comparison against substances with well‐quantified degradation profiles; (3) analytical methods that allow quantification for parent and metabolites at environmentally relevant concentrations, and inform on test substance bioavailability, biochemical pathways, rates of primary versus overall degradation, and rates of metabolite formation and decay; (4) modeling tools that predict the likelihood of microbial biotransformation, as well as biochemical pathways; and (5) modeling approaches that allow for derivation of more generally applicable biotransformation rate constants, by accounting for physical and/or chemical processes and test system design when evaluating test data. We also identify that, while such advancements could improve the certainty and accuracy of persistence assessments, the mechanisms and processes by which they are translated into regulatory practice and development of new OECD test guidelines need improving and accelerating. Where uncertainty remains, holistic weight of evidence approaches may be required to accurately assess the persistence of chemicals. *Integr Environ Assess Manag* 2022;18:1454–1487. © 2022 The Authors. *Integrated Environmental Assessment and Management* published by Wiley Periodicals LLC on behalf of Society of Environmental Toxicology & Chemistry (SETAC).

## Key points

- This critical review addresses the challenges and limitations facing current test methods used in persistence assessment and the scientific advances that are helping to both understand and provide solutions to them.
- Robust methods are now available to provide a deeper understanding of microbial composition, diversity, and abundance, ensuring consistency and/or interpret variability between tests; and quantify parent and/or metabolites at low concentrations informing bioavailability and biotransformation rates/paths.
- Benchmarking tools and reference substances are now available that aid in persistence evaluations through comparison against substances with well‐quantified degradation profiles.
- Modeling tools are available that predict the likelihood of microbial biotransformation and biochemical pathways, and allow for derivation of more applicable biotransformation rate constants, by accounting for physical and/or chemical processes and test system design when evaluating test data.

## INTRODUCTION

Chemical persistence in the environment is one of the most important criteria in the international regulation of organic chemicals (Cousins et al., 2019). Besides being used for prioritizing hazardous chemical substances, it is central to determining chemical exposure and subsequent risk to biota. Chemical pollution is one of nine identified factors that threaten to destabilize our Earth system processes (Rockstrom et al., 2009). The effects of exposure to anthropogenic chemical substances (henceforth, referred to as “substances”) are thus one of humanity's greatest challenges (Schwarzenbach et al., 2010). In this context, persistence has been proposed as a central, though not unique, indicator to help quantify boundaries for different substances (Diamond et al., 2015; MacLeod et al., 2014; Persson et al., 2013) in defining a “safe operating space for humanity” (Rockstrom et al., 2009). High persistence alone has even been suggested as a sufficient criterion for the regulation of substances of very high concern (SVHC) in the so‐called “persistence‐sufficient approach” (Cousins et al., 2019).

The conceptualization and importance of persistence have long been recognized and established (see Stephenson, 1977). For this paper, persistence is defined as the propensity of a substance to remain in the environment before being transformed by chemical and/or biological processes, whatever the emission compartment (e.g., air, water, soil, or sediment). In regulatory persistence assessment regimes, microbially mediated transformation processes are considered most important, or central, as microorganisms are ubiquitous and therefore impact the fate of substances in many environments (Figure S2, Tables S1 and S2). These will be the focus of the present paper.

Persistence may be assessed by laboratory and field studies, environmental monitoring, and computational modeling. In regulatory frameworks, the definition of persistence is operational; it is legally defined using threshold compartment‐specific half‐life criteria {e.g., Annex XIII of REACH [Registration, Evaluation, Authorisation and Restriction of Chemicals; Regulation (EC) No 1907/2006] and Annex II of (EC) No 1107/2009}. These half‐lives can be determined directly from laboratory simulation studies (OECD test guideline [TG] 307 [OECD, 2002a], OECD TG 308 [OECD, 2002b], OECD TG 309 [OECD, 2004a]). Under REACH, the integrated testing strategy (ITS) framework enables step‐wise decisions on whether a substance is not persistent, potentially persistent, or persistent, utilizing laboratory screening studies (see Supporting Information, *Persistence assessment—data interpretation and evidence*) as a first tier of tests (OECD TG 301 [OECD, 1992a], OECD TG 306 [OECD, 1992b], and OECD TG 310 [OECD, 2014]). For plant protection products in the EU, simulation studies are mandatory and half‐lives are determined for more than one environmental compartment using laboratory and field studies (EFSA, 2014). Although regulatory frameworks can differ in (i) the compartment‐specific persistence threshold criteria used and (ii) the approaches applied to identify and prioritize persistent substances, there are common features to the procedures involved (Boethling et al., 2009; Matthies et al., 2016). This includes the reliance of all frameworks on laboratory‐determined half‐life data or the ability to extrapolate and interpret data toward the legally defined thresholds. Major technical challenges are often encountered during laboratory testing, which can cause difficulties in drawing reliable conclusions on persistence.

Current persistence assessments evolved around tests that were originally developed >15 years ago (and in most cases, >30 years ago) based on the scientific evidence at the time. Some tests, such as the ready biodegradability tests (RBTs), were not specifically developed for the purpose of screening for persistent substances (Kowalczyk et al., 2015), but to identify substances undergoing rapid and ultimate biodegradation under environmental conditions. These laboratory tests are mostly suitable for water‐soluble, nonvolatile, and nonsorptive substances delivered as single constituents, not adequately reflecting the wide range of manufactured substances that find their way into the environment (including multiconstituent substances and polymers [ECETOC, 2019, 2020]). Furthermore, persistence is not a single fixed physico‐chemical property, but a manifestation of complex processes, a function of intrinsic substance properties and environmental conditions, which can change temporally and spatially (Fenner et al., 2004; McLachlan et al., 2017). A single test under specific experimental conditions testing a single constituent substance therefore cannot sufficiently reflect all environmental conditions in which substances are released. The rate of biotic and abiotic transformations differs depending on the environmental compartment and the physico‐chemical conditions within the environment. All these factors can lead to variability in substance half‐life estimates and hence uncertainty in the designation of persistence or nonpersistence.

In 2019, the European Centre for Ecotoxicology and Toxicology of Chemicals (ECETOC) set up a task force to evaluate and report on the scientific challenges and advances in persistence assessment since its last review 18 years ago (ECETOC, 2003). Some of the challenges identified then are still germane. The ECETOC task force “Moving persistence (P) assessments into the 21st Century” has formulated recommendations to improve P assessment. This paper addresses those scientific limitations, challenges, and opportunities related to improving the accuracy, reliability, and interpretability of laboratory methods to determine persistence half‐lives, particularly with respect to their relevance to the real environment.

The generation of robust half‐life data is just one, albeit important, step in regulatory hazard and risk assessment. However, environmental persistence and exposure is governed and influenced by many other factors. Many of these additional considerations are discussed in a separate companion paper by the ECETOC task force. It reports on the need for a clear and consistent weight of evidence framework, considering data in a multimedia context that includes the concept of overall persistence (*P*_ov_) (Redman et al., 2021).

In the following, the discussion of scientific challenges and progress is divided into challenges pertaining to the microbiology, obstacles in testing the degradability of substances, consideration of other abiotic processes, and how to link laboratory test outcomes to field monitoring data. We additionally highlight how modeling as a tool can be helpful in finding solutions to these challenges. Finally, we evaluate the state of science and its translation and reassessment in the context of regulation.

## MAJOR CHALLENGES BY THEME

### Relevance of microbial source, sampling, and sample treatment with respect to environmental conditions in the context of reducing test variability

Biodegradation tests form the basis of regulatory persistence assessments, and, as biocatalysts, microorganisms form the basis of the biodegradation tests. Typically, a given substance is only evaluated once by screening or simulation tests, even though the results of standard biodegradation tests are known to be highly variable. The inter‐ and intra‐laboratory variabilities of screening test outcomes are well documented (see Kowalczyk et al., 2015 for a review). The half‐lives of many substances are known to vary widely, sometimes by orders of magnitude (Birch, Hammershoj, et al., 2017; Latino et al., 2017; Seller et al., 2020). This causes difficulty in categorizing substances as persistent, especially if the variation in half‐lives ranges across the persistence threshold values. It also adds uncertainty to exposure and risk assessments for which half‐life data are also used.

The variations in biodegradation test outcomes have generally been attributed to variations in the so‐called “quantity and quality” of the natural microbial communities used as “inocula” in the tests (Birch, Hammershoj, et al., 2017; Forney et al., 2001; Goodhead et al., 2014; Honti et al., 2016; Martin et al., 2018; Ott, Martin, Acharya, et al., 2020; Shrestha et al., 2016; Thouand et al., 1995). “Quantity” typically refers to the cell *concentration* and/or the *total* amount of the inoculum or natural sample used in the test (determined by the source and volume of the test vessel) and “quality” refers to the microbial *community composition* and its activity, that is, the presence and viability of specific degrader taxa and the taxonomic and functional diversity and activity of the inoculum or sample. In some tests, the variation may also be attributed to differences in physico‐chemical conditions of the test or transport and availability issues of the substance (see other sections below).

Below, we highlight three aspects related to microbial biomass “quantity and quality” that have been repeatedly shown to cause variability in biodegradation test outcomes.

#### Is the microbial sample size in tests representative of the source environment?

**Figure 1:**
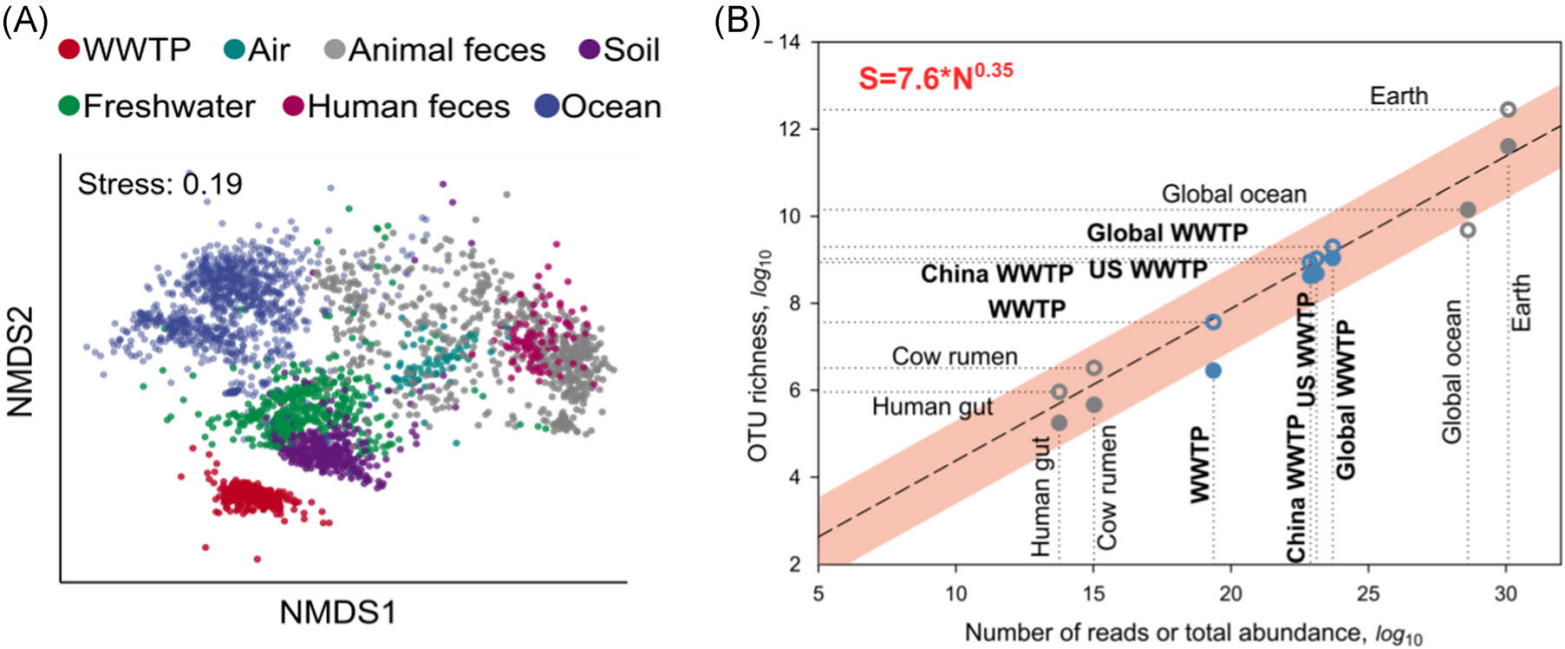
(A) A nonmetric multidimensional scaling (NMDS) ordination plot showing the similarity between microbial communities from different environmental source communities (colored data points), where the distance between the data points in multidimensional space reflects the similarity between communities (only shown in two dimensions here; NMDS1 and NMDS2), and (B) bacterial species richness (S) scaling with increasing sample size (in terms of the total cell numbers or total number of sequence reads, N), from individual samples to those estimated for the entire globe for different microbiomes (Wu et al., 2019). Reprinted by permission from Springer Nature: Nature Microbiology, Global diversity and biogeography of bacterial communities in wastewater treatment plants, Wu et al. (2019)

Implicit in all biodegradation tests is the assumption that a relatively small sample of any given environmental compartment is representative of the metabolic potential that a substance is likely to encounter in that compartment. There are an estimated 10^30^ prokaryotes on planet Earth, having evolved to a diversity of at least thousands (Amann & Rossello‐Mora, 2016), and controversially estimated at 10^12^ “species” (Locey & Lennon, 2016). It is thus questionable that the relatively small sample sizes used for biodegradation tests are sufficient to represent the microbial community encountered by substances freely diffusing throughout a given environment (Vazquez‐Rodriguez et al., 2003). For example, the inoculum in an RBT (~10^4^ cells/mL) is typically ten thousand times less concentrated than the same community in a typical activated sludge wastewater treatment plant (WWTP) (10^8^ cells/mL; Table 1), and the total number of cells in an RBT test vessel (10^8^) is a billion times fewer than that of a small‐sized WWTP (~10^17^ in 100 m^3^ reactor), with ensuing differences in diversity (*cf* section on *The under‐representation of microbial diversity in tests* and Figure 1). Across different biodegradation test systems, the cell concentrations (10^1^ cells/mL in some RBTs to 10^10^ cells/g in soil simulation tests) and the total number of microorganisms vary widely (10^5^ to 10^10^; Tables 1 and 2).

**Table 1:** Characteristics of the source, concentrations, total number, and pretreatment of sampled microorganisms and the substrate suitability for OECD screening tests (adapted from OECD, 1992a)

	Ready biodegradability							Biodegradability in seawater	Inherent biodegradability	
OECD test designation	301 A	301 B	301 C	301 D	301 E	301 F	310	306	302 B	302 C
Name	DOC Die Away	CO_2_ Evolution	MITI (I)	Closed Bottle	Modified OECD Screening	Manometric Respirometry	Headspace test	Biodegradability in seawater	Zahn Wellens/EMPA	MITI (II)
*Quantity of microbial source material*										
mg/L suspended solids	≤30	≤30	30	n/a	n/a	≤30	4 (≤30)	n/a	200–1000	30
mL effluent added/L	≤100	≤100	n/a	≤0.5	≤0.5	≤100	1–10	n/a	n/a	n/a
Typical test volume (L)	<1	2–3	<0.3	<0.3	<1	Not specified	0.125	<1	1–5 (typically 2)	0.3
Approx. cells/mL	10^4^–10^5^	10^4^–10^5^	10^4^–10^5^	10^1^–10^3^	10^2^	10^4^–10^5^	10^4^–10^5^	10^5^–10^7^	>10^5^	>10^5^
Max. total number of cells	10^8^	10^8^	10^7^	10^5^	10^5^	10^7^	10^7^	10^10^	5 × 10^8^	10^7^
*Source of microbes* a (cell concentrations)	Inoculum^a^	Inoculum	Inoculum	Inoculum	Inoculum	Inoculum	Inoculum	Natural^a^	Inoculum	Inoculum
(i) Activated sludge (10^8^– 10^9^ cells/mL)^b^	✓	✓				✓	✓		✓	
(ii) Secondary effluent (10^7^– 10^8^ cells/mL)^c^	✓	✓		✓	✓	✓	✓			
(iii) Surface water (10^5^–10^7^cells/mL)^d^,^e^	✓	✓		✓	✓	✓	✓	✓		
(iv) Soil (10^10^ cells/g)^f^	✓	✓	✓			✓	✓			
(v) Mixture of above		✓					✓		✓	✓
(vi) Sediment (10^8^ cells/mL)^d^										
*Pretreatment options*										
(i) Settling	✓	✓		✓	✓	✓			✓	
(ii) Filtering^g^	✓^fs,fp^	✓^fs,fp^		✓^fp^	✓^fp^	✓^fs,fp^				
(iii) Preconditioning^h^, ^i^	✓^a^	✓^a^	✓^b^	✓^a^	✓^a^	✓^a^				✓^b^
*Substance suitability*										
Poorly soluble	−	+	+	±	−	+	±		−	+
Volatile	−	−	±	+	−	±	±		−	−
Adsorbing	±	+	+	+	±	+	+		±	+

Abbreviation: DOC, dissolved organic carbon.

^a^ “Inoculum” represents an environmental source of microorganisms that is added (inoculated) into a buffered medium or solution in the test, whereas “natural” represents an environmental source of microorganisms without any additional buffered medium or solution in the test.

^b^ Brown et al. (2019).

^c^ Goodhead et al. (2014).

^d^ Whitman et al. (1998).

^e^ Ott, Martin, Acharya, et al. (2020).

^f^ Raynaud and Nunan (2014).

^g^ For 30 min or 1 h through a fine sieve (fs) or course filter paper (fp).

^h^ Preconditioning consists of aerating the inoculum (in mineral medium) for 5–7 days at the test temperature, but does not allow preadaptation to the test substance.

^i^ Preconditioning consists of mixing inocula, pH adjustment, allowing them to stand, adding the supernatant of filtered activated sludge, and aerating the suspension for between 1 and 4 months along with replacement of a third of the settled supernatant with a glucose–peptone–phosphate medium every 24 h.

Box 1 Biological diversity is comprised of three types: alpha diversity, which is the taxa richness (number of different types) and/or their abundance distribution (evenness) within a given community; beta diversity, which is the difference in taxa (types and abundances) between different communities; and gamma diversity, the total diversity of taxa in a landscape (Magurran, 2004). The taxon of interest in ecology is usually the “species,” for which there is no strict definition for uncultured microorganisms (which are the overwhelming majority), but “species” are often defined by sequence dissimilarity cut‐off (often >3%) in their 16S rRNA gene, a universal phylogenetic marker (Stackebrandt & Goebel, 1994).

**Table 2:** Characteristics of the source, concentrations, and total number of sampled microorganisms and the substrate suitability for OECD simulation tests

	Simulation tests		
OECD test designation	307	308	309
Name	Aerobic and anaerobic transformation in soil	Aerobic and anaerobic transformation in aquatic sediment systems	Aerobic mineralization in surface water simulation biodegradation test
*Amount of microbes*			
mg/L suspended solids	50–200 g dry wt	>50 g sediment/>3× volume water	>100 mL; 165–333 mL
mL effluent added/L	n/a	n/a	n/a
Typical test volume (L)	Not specified	Not specified	0.5–1
Approx. cells/mL or g	10^10^	10^5^–10^8^	10^4^–10^7^
Max. total number of cells	10^12^	10^9^–10^10^	10^9^
*Source of microbes* *(cell concentrations)*	Natural	Natural	Natural
(i) Surface water (10^5^–10^7^cells/mL)^a^, ^b^		✓	✓
(ii) Soil (10^10^ cells/g)	✓		
(iii) Sediment (10^8^ cells/mL)^a^		✓	(✓)
*Substance suitability*			
Poorly soluble	+	+	±
Volatile	−	±	±
Adsorbing	±	±	±

^a^ Whitman et al. (1998).

^b^ Ott, Martin, Acharya, et al. (2020).

The OECD TG 309 surface water simulation test can be conducted with surface water only (pelagic test, preferred in REACH, ECHA, 2017a) or amended with sediment to represent water bodies with suspended solids (suspended sediment test). Variations in cell concentrations in natural surface waters span three orders of magnitude (Tables 1 and 2) depending on the type and location of the source, and the cell concentrations in sediments are significantly higher than those in surface waters (10^8^/mL and 10^6^/mL, respectively; Table 2). The amount of sediment added to the test is allowed to differ by two orders of magnitude (0.01–1 g/L). Recent data suggest that there are large uncertainties in degradation half‐lives derived from OECD TG 309 tests linked to the introduction of sediment and the amount of “active” biomass that they contain (Honti et al., 2016; Seller et al., 2020; Shrestha et al., 2016). Similarly, in RBTs, seven different types of test designs are widely used and inocula can be selected from five different environmental sources with different concentrations and microbial communities (Kowalczyk et al., 2015; OECD, 1992a, 2014). The inocula can further be prepared in up to four different ways (Table 1; Goodhead et al., 2014). Consequently, RBT outcomes for a given substance can differ widely (Goodhead et al., 2014; Thouand et al., 1995; Vazquez‐Rodriguez et al., 2003).

#### Under‐representation of microbial diversity in tests

Microbial communities are known to show both similarities and differences in their composition within and between environmental compartments (Thompson et al., 2017; Wu et al., 2019) (Figure 1A). However, regulatory persistence assessments tend to prioritize the aquatic environment (wastewaters for RBTs, fresh, or marine water for simulation tests) unless this compartment is not considered relevant for emissions or persistence criteria are expected to be exceeded in sediment or soil. Current assessments are also mainly pelagic, while 40%–80% of microbial communities on Earth exist in biofilms (Flemming & Wuertz, 2019). RBTs typically use settled or filtered secondary effluent or activated sludge, and the OECD TG 309 uses freshwater or seawater, but can also incorporate sediments that can harbor and cause biofilm growth during the duration of the study (Tables 1 and 2). Anaerobic environments are not considered “to be especially relevant scenarios for the persistence assessment in the EU” (ECHA, 2017b), despite the majority of microbial life existing in the subsurface (Flemming & Wuertz, 2019; Whitman et al., 1998) and some form of anaerobic biodegradation tests being available that could be used or adapted for such purposes (e.g., OECD TG 307, 308 and 311 [OECD, 2006]). While plenty of evidence exists that the origin, size, and concentration of the microbial community used in biodegradation testing strongly influence biodegradation test outcomes (Birch, Hammershoj et al., 2018; Brillet et al., 2016; Goodhead et al., 2014; Martin et al., 2018; Ott, Martin, Snape, et al., 2020; Thouand et al., 1995), the underlying causal links are poorly understood.

The important role that microbial community diversity plays (see Box 1 for definition of biological diversity) in accounting for the variability observed in biodegradation tests is of increasing interest. Fundamentally, there is a relationship between sample size and/or concentration and the number of different taxa (e.g., “species”) in a sample (Martin et al., 2018). Given the small sample sizes and low cell concentrations used in most biodegradation tests, the sampled diversity is a negligible fraction of the community diversity from which they are taken (Figure 1B).

Consequently, some catabolic functions and community networks (i.e., ecological networks of co‐occurring taxa showing parasitism, commensalism, mutualism, amensalism, or competition) present in a real environmental system may rarely be sampled into biodegradation tests. The resulting chance inclusion or exclusion of specific degraders (i.e., those microorganisms harboring the necessary metabolic potential to transform a given substance) is postulated to explain the high variability observed in RBTs, a phenomenon termed the “biodegradation lottery” (Martin, Snape, et al., 2017; Ott et al., 2019).

In this context, it is worth noting that, under REACH, substances failing an RBT require further, more expensive simulation tests, and those that additionally have a log *K*_*ow*_ (n‐octanol‐water partition coefficient) >4.5 may also require bioaccumulation and toxicity tests at considerable cost and an estimated 600 fish per substance (Martin, Goodhead, et al., 2017).

#### Microbial adaptation to substances is poorly captured in tests

Microorganisms can undergo acclimation and adaptation in response to exposure to novel chemicals, which, in turn, may affect their persistence (Poursat et al., 2019). Although “adaptation” normally refers to genetic changes that result in new phenotypes, this term is often used in persistence testing to mean any change in a microbial community due to long‐term exposure to the test substance (OECD, 2006). A shift in the relative abundance of species in a microbial community in the presence of the test substance would be considered an adaptation, even if the genetic library composition of the community remains the same. Acclimation is the short‐term process by which a microbial inoculum adjusts to the test conditions, such as testing outside ambient conditions (e.g., at temperatures higher than those at which the environmental sample was sourced).

Adaptation to the presence of chemicals is a naturally occurring phenomenon that has been observed in almost all environmental compartments and can be induced in the laboratory. It is well documented in the literature and is commonly attributed to several key mechanisms including proliferation of competent degraders, gene transfer or mutation, induction of enzymes, or the change in other environmental factors affecting biodegradation. There are many known cases where enzymes have evolved to transform substances that were previously nonbiodegradable (Wackett & Robinson, 2020). Poursat et al. (2019) reviewed laboratory studies where adaptation was observed for 18 different substances covering a wide range of substance classes (e.g., dyes, herbicides, chelators, phenols, and quaternary ammonia compounds). Two particularly interesting cases are known where adaptation was observed in “real time” in the field. In one case, Itrich et al. (2015) showed that inocula from US WWTPs were unable to degrade l‐glutamate‐*N*,*N*‐diacetate (L‐GLDA) prior to its market introduction, whereas, within less than two years of the US market launch of this down‐the‐drain‐chemical, L‐GLDA went from failing all RBTs (with limited biodegradation observed) to passing RBTs with inocula from 12 different US WWTPs. At the same time, they also showed that WWTP inocula could be adapted in the laboratory under realistic environmental conditions (using OECD 303A WWTP simulation test systems to adapt inocula) to biodegrade L‐GLDA and pass RBTs (Itrich et al., 2015). In the second case, the sudden onset of biodegradation of the artificial sweetener acesulfame in full‐scale WWTPs globally has been demonstrated after years of limited to no biodegradation being observed (Kahl et al., 2018). These observations appear to support the notion of “microbial infallibility” (Kleinsteuber et al., 2019), which hypothesizes “that all organic compounds could be biodegraded if only the right organism could be found, the right enzymes induced, and the prevailing environmental and nutritional conditions for its growth on that substance were suitable” (Painter, 1974). However, they are mostly from situations with high potential for exposure to the respective chemical (i.e., WWTPs or agricultural soils). It thus remains to be explored whether the phenomenon of adaptation also takes place under conditions more closely resembling natural background levels. Also, the concept of microbial infallibility may not apply to all substances (e.g., highly fluorinated chemicals). Under REACH, acclimation is permitted, while adaptation is not (ECHA, 2017a). However, it is reasonable to expect that microbes in many compartments have been exposed to industrial and naturally occurring chemicals. It has been suggested that including acclimation and adaptation in biodegradability tests may also reduce the variability often observed in tests using different inocula (Dalmijn et al., 2020). Taking account of adaptation processes in simulation tests would more accurately test the inherent degradability of a substance (Table 3). Failure to take these processes into account may lead to overestimation of the persistence of many chemicals, but general agreement is required on how adaptation should be included in persistence testing.

**Table 3:** A list of innovations and techniques that could be considered for use in persistence assessments, the challenges and/or limitations that they tackle, how and where they could be used, and their advantages and disadvantages

Challenge and/or limitation addressed	Innovation or technique	Description	How and where they could be used	Advantages	Disadvantages
(1) High variability and high failure rate of nonpersistent chemicals in BSTs.	Improve representation of microbial cell numbers and diversity: A new marine biodegradation screening test for persistence assessment (MaP test) (Ott, Martin, Acharya, et al., 2020).	New marine biodegradation screening test with increased cell concentration shown to improve the probability of correctly classifying the biodegradation category of reference benchmark chemicals compared to the current OECD 306 test.	As an alternative or additional screening test in the ITS (see Supporting Information Section 2.4) for chemicals emitted into the marine environment.	Has been developed in accordance with OECD document 34 guidance on validating new tests (OECD, 2005). An estimated saving of $75K and 600 fish per chemical accurately identified as nonpersistent (see Section 2.1.2; Martin, Goodhead, et al., 2017). Accepted as an interim test for persistence assessment by OSPAR (OSPAR, 2021).	Not yet an accepted OECD test guideline. Contravenes existing ECHA REACH guidance by using increased cell concentrations in the test compared to existing tests (Ott, Martin, Snape, et al., 2020). Validated using relatively few soluble, nonvolatile chemicals.
(2) Ability to count total (and viable) bacterial cells from many different environmental matrices without culture bias. Could replace heterotrophic plate counts (only measures 0.01%–1% of the total cell counts [WHO, 2003]), total suspended or volatile solids, and/or COD or TOC measurements of biomass.	Flow cytometry.	A method to count total and viable cells using fluorescent DNA stains that is used extensively in microbiology.	In all biodegradation tests—at the beginning and end of a test.	Already accepted as a recognized standard test for water quality monitoring in Switzerland (Van Nevel et al., 2017). Existing methods published for use on marine, freshwater, and activated sludge matrices (Brown et al., 2019; Ott, Martin, Acharya, et al., 2020; Vignola et al., 2018). Can convert cell numbers into biomass and carbon estimates for mass balances. Can be used to normalize or standardize first‐order rates to biomass levels.	New to the chemical regulatory industry supply chain. Requires investment in new equipment, knowledge, and skills. Not standardized for all environmental matrices.
(3) Variation in biodegradation outcomes due to differences in the microbial community of the microbial sources used in biodegradation tests.	Molecular microbial ecology techniques such as high‐throughput next‐generation sequencing of markers for taxa (16S rRNA gene amplicon sequencing), functional genes (metagenomics), or their transcripts (metatranscriptomics).	Methods to identify taxa, their functional genes, and transcripts for expressed proteins or enzymes.	Provides information on the diversity and structure of communities from a given sample. Can be used to assess how similar different source samples are; investigate the relationship between diversity and substance degradation; and identify putative taxa and/or enzymes associated with substance degradation.	Provides information at an unprecedented scale and resolution that overcomes “cultivation bias.” Costs are decreasing, and the amount of data acquired is increasing, exponentially. Can provide added context to biodegradation anomalies and outcomes. Commercial services available.	Technically difficult to prepare (requires some expertise and specialized equipment). Methods not standardized. Requires expert data analysis to interpret results. Understanding of the theoretical ecological framework within which to interpret results still being developed.
(4) Adaptation not taken into account.	Include adaptation in simulation testing methods.	Extend test duration or use inocula pre‐exposed at environmentally relevant substance concentrations.	Simulation tests (and enhanced screening tests).	Provides more relevant information on the ability of microbes to degrade substances and reduces the variability of test outcomes.	Requires standardized approaches to be implemented. Contravenes existing ECHA REACH guidance that does not allow adaptation (e.g., pre‐exposure to substances).
(5) Sequestered and covalently bound nonextractable residues (NERs) can be of xenobiotic and biogenic nature. Xenobiotic residues can be analyzed by chromatographic methods, but a method to quantify biogenic residues experimentally in both types has not been developed yet. An estimate of total bioNER can be performed using the MTB modeling approach.	Silylation of NER contained in soil or sediment (Schäffer et al., 2018).	By silylation, a chemical derivatization method of humic matter in soil or sediment, sequestered and covalently bound NER can be distinguished.	Sequestered residues are slowly released from the matrix. If the parent or relevant metabolites have been analyzed in such (sequestered) residues, this can be considered in persistence assessment.	Silylation so far is a method to distinguish between sequestered and covalently bound residues, the latter being strongly bound with little release potential. The alternative extraction methodology using EDTA (ECETOC, 2013b) has the disadvantage that large parts of biogenic residues (proteins, nucleic acids, phospholipids, etc.) are extracted as well.	Method has so far been used for scientific research and needs validation, e.g., by ring testing. Development of a robust silylation method is under way (see text).
(6) Test substance toxic to microbes at test concentrations and/or test substance is not soluble at test concentrations.	(A) Passive dosing (Smith et al., 2012).	Sorb test substance to a passive dosing matrix and dose the test system accordingly. Test substance will desorb depending on the sorbent and the test substance. Prework will be needed to identify the correct sorbent.	In any test system where a constant low‐level concentration of test substance is needed. Especially important when the test substance is toxic at test concentrations.	Allows for microbial exposure to a constant low‐level concentration of the test substance in the test system.	The rate of biodegradation is slower as first; the test substance must desorb from the sorbent. An additional phase (sorbent) is added to the test system so care must be taken to ensure that it does not negatively impact test results. The potential formation of monoculture algal overgrowth.
	(B) Biodegradation kinetic testing of mixtures at low environmentally relevant concentrations (Hammershøj et al., 2019).	Biodegradation experiments are conducted at low concentrations in gas‐tight autosampler vials, and Solid Phase Microextraction coupled to GC‐MS is then used to determine substrate depletion relative to abiotic controls. Passive dosing can be used to set initial concentrations without the addition of a co‐solvent.	For the testing of chemicals in mixtures.	Simultaneous generation of biodegradation kinetic data for a large number of chemicals. The method yields well‐aligned and better comparable data, since many chemicals are tested with the same inoculum.	Limited to “primary degradation.”
(7) Persistence assessment of substances with low bioavailability in soil and sediment.	Bioavailability‐based persistence assessment.	Base persistence assessment on the bioavailable fraction of substances in soil and sediment tests.	Higher‐tier tests (OECD TG 307, 308).	Clarifies the impact of bioavailability on the persistence of substances.	Standardized methods for bioavailability correction need to be developed and implemented.
(8) Artifacts due to testing at unrealistically high concentrations and lack of information on substances showing slow mineralization.	Primary degradation‐based persistence assessment. Passive dosing (see 6A).	Testing at environmentally relevant concentrations using specific analyses to determine primary (bio)degradation in combination with nonspecific measurement of mineralization and nontarget analysis to identify metabolites.	Screening and higher‐tier tests.	Avoids potential artifacts due to slow dissolution and toxicity. Generates more relevant data for effects assessment. (e.g., consideration of relevant metabolites in assessment).	Methods need to be developed and implemented.
(9) Holistic understanding of the potential for abiotic degradation in aquatic environments.	Increased use of indirect photolysis studies.	As these studies (using natural waters) are not mandatory, they are rarely performed. They capture the potential for photodegradation sensitized by natural constituents of surface waters (N, DOM, etc.).	In the first tier of testing abiotic factors, alongside hydrolysis and direct photolysis studies.	Established guideline for direct photolysis exists (OECD TG 316). The methodology for indirect photolysis is the same, but using actual or simulated natural water (containing photosensitizers) instead of buffer. Research has shown that indirect photolysis can be a significant process for compounds that do not directly absorb UV light.	Careful selection and justification of the natural water used will be required as the results will be dependent on the concentrations of photosensitizing species in the water.
(10) Lack of understanding of the combined effect of biotic and abiotic processes.	Implementation of the irradiated water‐sediment study.	Higher‐tier optional study in pesticide data requirements that combines the OECD308 simulation study with an indirect photolysis study (i.e., water‐sediment systems irradiated by a xenon‐arc lamp or sunlight).	Either to replace the existing OECD TG 308 study or as a higher‐tier option where standard simulation studies (OECD TG 308 and 309) indicate persistence.	Established methodology that can easily be implemented in existing testing laboratories that have the capability to perform photolysis studies.	No formal guideline exists for the study (however, the requirements of OECD TG 308 and 316 are combined).
(11) Conducting studies in the dark does not capture the biodegradation potential of all microorganisms as phototrophs will be dormant. The indirect impact of the growth of algal communities on heterotrophic communities is also not captured.	Conducting simulation studies under non‐UV light.	Any simulation study can be conducted with a light–dark cycle of non‐UV (fluorescent light). This allows active photosynthesis of indigenous algal populations, and their metabolic contribution can be captured.	Either to replace the existing OECD TG 308 study or as a higher‐tier option where standard simulation studies (OECD TG 307, 308, and 309) indicate persistence.	The existing OECD TG 309 guidelines allow for such a modification (diffuse light). OECD307 and 308 studies can be easily modified by utilizing lighting systems commonly used for algal ecotoxicology studies. The use of non‐UV light allows for discrimination between algal metabolism and photolysis.	No formal guidelines exist for the OECD TG 307 and 308 studies to be modified in this way. For OECD TG 309, there is no clear definition of diffuse light, but fluorescent light as used in ecotoxicology studies can be considered diffuse.
(12) Total system degradation half‐lives from simulation studies (e.g., OECD 307 or 308) are affected by phase transfer, NER formation, and biomass concentration, and hence are highly variable across systems and environmental matrices.	Use inverse modeling to derive a bioavailability‐ and biomass‐normalized k'bio value (i.e., biomass‐corrected pseudo‐first‐order biotransformation rates).	Simulation study systems are represented as a combination of solid and aqueous phases connected by phase‐transfer processes, and a sequence of transformation processes taking place in the aqueous phase (Honti et al., 2016). Given measured concentrations, k'bio can be estimated through inverse modeling of this system.	k'bio can be used directly in exposure modeling to account for varying extents of bioavailability and biomass density between environmental compartments.	k'bio describes the intrinsic biotransformation potential of a compound. As such, it facilitates comparison of biotransformation between different test systems or test systems and *in‐field* behavior (Honti et al., 2018).	k'bio values are not directly interpretable, and cannot be considered persistence indicators per se. They would need to be back‐converted into persistence half‐lives using a number of assumptions on typical solid‐to‐water ratios and biomass concentrations in a given environmental compartment of interest.
(13) Need to develop models for predicting persistence in different environmental compartments, but lack of sufficiently large and consistent data sets for model development	Use of different normalization techniques to merge data into larger data sets.	Normalization of biotransformation half‐lives can be achieved by correcting for processes that are reasonably well understood (i.e., bioavailability, temperature, biomass) or they can be calibrated relative to the behavior of one or several benchmark chemicals (McLachlan et al., 2017).	Larger data sets can be used to develop quantitative–structure–biotransformation relationships or to develop read‐across approaches to predict half‐lives in one compartment from those in another compartment (Fenner et al., 2020).	Ability to perform hazard and risk assessment much more broadly across substances on the market, but also during the substance development phase in industrial research.	The size of available data sets is still limited. Some mechanistic process understanding is mandatory to develop models on these still limited data sets.

*Note*: Refer to the main text in *Current and future options for persistence assessment* for further explanations and references to the innovations outlined.

Abbreviations: BST, biodegradation screening test; COD, chemical oxygen demand; DOM, dissolved organic matter; EDTA, ethylenediaminetetraacetic acid; MTB, microbial turnover to biomass; N, nitrogen; NER, nonextractable residues; TOC, total organic carbon.

### Obstacles with test substances

#### Volatile substances

Biodegradation mainly occurs in aqueous or moistened environments (Kästner et al., 2014), but the importance of interstitial air as a mass transfer medium for semivolatile chemicals has also been highlighted. This is because water is the exclusive transport medium for the substance to encounter a metabolizing agent (e.g., enzyme). For substances that strongly partition to air from water, there is a competition between the rate of encounter between the substance and the metabolizing agent (biodegradation) and the rate at which the substances diffuse from water into air (volatilization). When the volatilization rate is not negligible compared to the biodegradation rate, technical issues arise in biodegradation tests because the substance disappears from the water phase before it has a chance to be biodegraded. Equilibrium between water and air is never reached in an open system since air plays the role of an infinite sink, resulting in a continuous loss of the test substance. There is as yet no validated regulatory protocol that can be used as a routine method to evaluate biodegradation rates of substances with nonnegligible volatilization rates. Currently, a common approach to address volatile losses in biodegradation experiments is to include abiotic controls, which can be used to correct observed disappearance for volatile losses, leaving the remaining loss attributable to biodegradation. However, this approach can overestimate volatile losses. In some cases, absorbent traps are used to capture volatile substances and maintain mass balance. The use of closed systems is often suggested in standard guidelines but has also been criticized (e.g., OECD TG 309) since such systems can make it difficult to maintain aerobic conditions (Shrestha et al., 2019). For the soil compartment, Shrestha et al. (2019) carried out a proof‐of‐concept study addressing the feasibility of OECD TG 307 simulation tests for two volatile hydrocarbons (tetralin and decane). For the sediment compartment (OECD TG 308), Shrestha et al. (2020) were unable to develop an appropriate protocol with the same substance despite numerous trials. For the water compartment, biodegradation testing in gas‐tight autosampler vials has recently been shown to be effective for the testing of semivolatile and volatile substances (Birch et al., 2018). Testing in closed vials does not only minimize evaporative losses but also allows biodegradation kinetics to be corrected for headspace partitioning (Birch, Andersen, et al., 2017).

#### Poorly water‐soluble substances

Difficulties encountered in estimating the biodegradability of poorly water‐soluble substances are often linked to their aqueous solubility and limited bioavailability to microorganisms (Alexander, 1999; Stucki & Alexander, 1987). Laboratory tests according to OECD or International Organization for Standardization (ISO) guidelines prescribe, in many cases, test substance concentrations well above the solubility limit for poorly soluble substances, since testing in the ng/L to µg/L range is experimentally challenging for many approaches (Sweetlove, 2017). However, operating biodegradation tests near or above the solubility limit can lead to an underestimation of biodegradability when dissolution of the chemical becomes rate‐limiting or when high test concentrations inhibit the biodegradation process (Hammershøj et al., 2019, 2020).

In soil and sediment tests, the bioavailability and, thus, biodegradability (Semple et al., 2004, 2007) of poorly soluble substances are limited due to their high affinity to solid matrices. Research performed during the last 30 years on organic chemicals has shown that estimating their biodegradability in soil and sediment based on total concentrations without accounting for their bioavailability may lead to wrong assessment of the persistence and overestimation of the environmental risks of poorly soluble substances. Examples of the application of the bioavailability of organic substances fall in the domain of retrospective risk assessment, that is, the management and remediation of polluted sites (Burkhard & Mount, 2017), but this is largely unexplored in prospective risk assessment (pRA) such as in REACH (Ortega‐Calvo et al., 2015). A remarkable example is phenanthrene, recently confirmed to be a substance of very high concern (SVHC) because it might be very persistent and very bioaccumulative (vPvB) (ECHA, 2018). The SVHC draft decision reasoned that phenanthrene is vP in soil, despite conflicts between the biodegradation rate and bioavailability of this substance (Hughes et al., 2020); for example, bioavailability may be reduced when the substance is strongly sorbed to organic matter of soils or sediments. By providing a more accurate reflection of the intrinsic properties of substances, bioavailability science is ready to improve the realism of the persistence assessment of poorly soluble organic substances, but there is a clear need to implement this knowledge in currently available methodologies.

Passive dosing uses a polymer loaded with the test substance as a donor to provide better defined concentrations of poorly soluble test substances in various types of tests. Passive dosing is increasingly being applied to biodegradation tests of poorly soluble test substances, which can be done in two fundamentally different ways. One approach is to include the loaded polymer in the biodegradation tests for the continuous release of test substances (i.e., dynamic passive dosing) (Smith et al., 2012), which has some resemblance to the chemodynamics of these substances in soils and sediments. Another more recent approach is to apply passive dosing to set initial concentrations of poorly water‐soluble substances in aquatic media, but without including the passive dosing donor in the test (Birch, Andersen, et al., 2017; Hammershøj et al., 2019, 2020). Hammershøj et al. (2019) applied this approach to test the biodegradation of hydrophobic substances in mixtures while varying the test substance concentration (ng/L–μg/L) and the number of mixture components. Interestingly, they observed longer half‐lives for single substances when tested at higher concentrations that approached aqueous solubility. This was also shown in biodegradation tests with lavender oil using surface water from a rural stream as the inoculum: delayed biodegradation kinetics at high concentrations was best explained by mixture toxicity near the aqueous solubility limit (Hammershøj et al., 2020). These approaches facilitate the testing of poorly water‐soluble substances at environmentally relevant low concentrations, while minimizing losses. However, they require analytical methods and instruments that are suited to measure substrate depletion well below the aqueous solubility of the given test substance.

#### Nonextractable residues (NERs)

Most chemical substances in soils and sediments form so‐called NERs, besides extractable and volatile residues (Barriuso et al., 2008; Kästner et al., 2014). This is observed in OECD test systems (OECD 307, 308, 309) and also in plant and animal studies. The formation of NERs hampers the determination of biodegradation rates, which were so far calculated from the rates of substance transformation derived from analysis of “extractable” residues and NER. For persistence assessment, the definition of NER is operational and based on methodological approaches.

Nonextractable residues are those substances retained in a matrix after exhaustive extractions that do not significantly transform the physico‐chemical structure of the solid. To obtain a matrix containing only NER, as a first step, the matrix (soil, sediment, plants, animal tissue) has to be thoroughly extracted. A proposed extraction sequence comprises aqueous solutions to determine the bioavailable residues being easily desorbed, followed by the use of organic solvent mixtures to extract the matrix efficiently, and finally, exhaustive extraction methods like Soxhlet or pressurized liquid extraction (PLE) or accelerated solvent extraction. The importance of selecting an appropriate extraction procedure was recently highlighted (Loeffler et al., 2020; Schäffer et al., 2018), with PLE and a ternary solvent mixture (methanol/acetone/water, 50/25/25, v/v/v) being, in many cases, the most suitable one, although variations in the extraction solvents according to the chemical properties of the test substance can lead to higher extraction efficiencies. The extraction scheme summarized in ECETOC Technical Report 117 includes the use of the chelating agent ethylenediaminetetraacetic acid (EDTA) (ECETOC, 2013a). The EDTA will lead to disaggregation of soil organic matter (SOM) and the partial release of NERs by chelating bivalent metal ions like Ca^2+^ that can form a variety of binding interactions leading to SOM aggregation.

After thorough extraction, the resulting matrix is assumed to contain only NER. Qualitatively, NERs can be categorized according to their nature, for example, type 1 (entrapped or sequestered), 2 (covalently bound), and 3 (biogenic) NER (Kästner et al., 2014). Two methods and definitions for differentiation of these three NER types have been proposed (ECETOC, 2013a; Schäffer et al., 2018) (see *Characterization of NERs*). Isotope‐labeled substances have been used for NER characterization, preferably with ^14^C, but stable isotopes like ^13^C can also be used (Kästner et al., 2016; Nowak et al., 2018), although for the latter, higher concentrations must be used for analytical reasons. The immobile (covalently bound) fraction 2, which is associated with the soil matrix, can be quantified using established wet‐chemistry techniques in combination with nonspecific analysis of the total radioactivity in each generated fraction (Mamy et al., 2015), but more specific derivatization methods such as silylation to further characterize NERs still need validation (see *Characterization of NERs*).

Besides methodological challenges involved in its analysis, NERs are considered in the risk assessment in varying ways, depending on the regulatory framework. Nonextractable residues are considered either to be reversibly bound to the soil or sediment and to pose a potential risk to the environment, or to be irreversibly bound and/or transformed, in part into biomass, which can be interpreted as a safe sink. To improve and consolidate the risk assessment regarding NER, a proposal for a tiered approach was made (ECHA, 2019).

The residues associated with NER transform and degrade at a different (slower) rate compared to the “extractable” fraction (Schäffer et al., 2015). If only minimal characterization of the NER fraction is performed, a conservative approach was suggested (ECHA, 2019) that considers all radioactivity associated with this fraction to be the parent substance. In that case, NER has to be added to the parent compound pool in the derivation of degradation half‐lives. However, if NER is further characterized, that is, differentiating types 1, 2, and 3, the above conservative approach does not need to be used and only type 1 residues have to be considered alongside the parent compound in persistence assessment, unless there are indications that type 2 residues are mobilizable (ECHA, 2019; Schäffer et al., 2018).

#### Testing persistence at environmentally relevant low substance concentrations

Many biological processes are concentration dependent, which requires careful selection of the substrate concentrations used in persistence assessment to understand environmental processes. Substances need to be tested at concentrations that predict behavior in the environment, and yet are high enough for the detection of biodegradation, while not so high as to induce toxicity to the microbes. Concentrations of substances in the environment are often low, typically well below their solubility threshold (Gobas et al., 2018). Therefore, persistence testing should be designed to characterize the behavior at concentrations that are representative of the exposure scenario. However, the design of RBTs usually does not allow for testing substances at µg/L concentrations or lower as biodegradation is measured indirectly through quantification of generic parameters (O_2_, CO_2_, dissolved organic carbon [DOC]), requiring substance concentrations in the range of 1–100 mg/L. Yet, testing at such high concentrations is experimentally challenging and can involve the need to use emulsifiers, solvents, and carriers to achieve reliable exposure concentrations. This helps to avoid dissolution that may otherwise limit biodegradation rates. These approaches may, however, cause unintended artifacts, for example, O_2_ consumption and CO_2_ production, and may even induce anoxia due to the degradation of the relatively higher concentrations of biodegradable solvents or emulsifiers (Shrestha et al., 2019). In addition, high test concentrations in RBTs can cause microbial inhibition for some test substances, and lower test concentrations are therefore needed to overcome this issue as discussed in the OECD301 TG. In addition, some chemicals are not soluble in RBTs due to the high test substance concentrations, but they are soluble at the lower concentrations present in the actual environment. Lack of solubility in an RBT impacts the bioavailability of test substances to the microbes and therefore impacts biodegradation. Considerable progress has been made in the last few years with regard to aligning biodegradation kinetic testing with modern gas chromatography‐mass spectrometry (GC‐MS) and liquid chromatography‐mass spectrometry (LC‐MS) analytics, which allows biodegradation kinetic testing of chemicals in mixtures at low environmentally relevant concentrations (Birch et al., 2018; Fenner et al., 2020).

#### Multiconstituent substances

Most screening biodegradation tests are conducted using single chemical substances at high concentrations, although these chemicals are often present in the environment as mixtures at low concentrations (Hammershøj et al., 2019).

This is because in screening tests, it is difficult to interpret the biodegradability result obtained for a multiconstituent substance with structurally dissimilar constituents when one or more constituents may be biodegradable. Testing each constituent of such a substance appears to be a possibility, but involves various constraints:

1. Multiconstituent substances such as substances of unknown or variable composition, complex reaction product, or biological material (UVCBs) do not necessarily have a defined composition.
2. It is sometimes impossible to obtain each constituent individually (e.g., essential oils) because they do not exist commercially or the constituent is not fractionable and extractable, or the individual constituents cannot be radiosynthesized.
3. Natural products may contain different constituents, and interactions of the individual substances may lead to solubilization or dispersion of some constituents, improving their accessibility to microorganisms (Auffret et al., 2009); on the contrary, such interactions can reduce the accessibility of some constituents (Bielefeldt & Stensel, 1999; Charng et al., 1993; Hammershøj et al., 2019).
4. It seems difficult or even unrealistic for private and public laboratories to assess up to hundreds of constituents to reach a conclusion on the biodegradability of a single complex substance.

In some cases, testing of such substances seems feasible; for example, for hydrocarbons, a class of UVCB with multiple constituents, the degradation of single substances does not appear to be different from the degradation of individual constituents in the mixture (Birch, Hammershøj, et al., 2017; Brown, Lyon, et al., 2020; Prosser et al., 2016). Multiconstituent substances also include polymers, though some polymers may not be multiconstituent substances. Polymers may be regarded as a class of their own apart from UVCBs. It is important to recognize that such high‐molecular‐weight substances will have additional different physico‐chemical properties and environmental behaviors that may require further modification of existing biodegradation testing methods (ECETOC, 2020).

#### Testing chemicals exerting toxicity for degrading microorganisms

Certain test substances, for example, some amine derivatives (van Ginkel et al., 2008) and cationic surfactants (Timmer et al., 2020), can inhibit microbial degradation in standard biodegradation tests by exerting toxicity on the microbial inoculum, especially at the mg/L concentrations applied in OECD biodegradation tests. Hammershøj and co‐workers have recently shown delayed biodegradation kinetics of hydrophobic petroleum hydrocarbons (Hammershøj et al., 2019) and the UVCB Lavender oil (Hammershøj et al., 2020), and explained this by substrate toxicity near the solubility limit. Substances with inhibitory effects should therefore be tested in an OECD TG 301 at 1/10th of the EC50 (concentration that affects 50% of a population) obtained in toxicity testing (OECD 209 TG), but this often leads to problems with detection limits using nonspecific analyses such as CO_2_ production and limits testing options.

As outlined in *Testing persistence at environmentally relevant low substance concentrations*, modern analytic techniques offer possibilities to avoid such problems.

### The effect of abiotic factors on persistence testing

The following sections review the impact of abiotic factors currently considered in the regulatory paradigm and for which standard test methods exist. This section is not intended as a full review of all potential abiotic factors that can occur in any biodegradation study or in the environment.

#### Hydrolysis and photolysis

Although the main focus in environmental persistence studies is on biodegradation, for some chemicals, hydrolysis (reactions with water) and photolysis (light‐catalyzed reactions) may contribute significantly to their environmental fate. Hydrolysis studies provide a direct measure of degradation rates when performed at environmentally relevant pH values. However, the pH at which hydrolysis can occur will depend on the structure of the substance, for example, esters may hydrolyze more rapidly under alkaline or acidic conditions. This leads to a complication in the assessment, as the environmental pH will vary, and hence the extent of hydrolysis will also vary (Katagi, 2002). This variation means that hydrolysis will only be a significant factor at the field scale, where hydrolysis is rapid at environmentally relevant pH values.

The relevance of photolytic processes in degradation, and hence persistence, will also depend greatly on the substance's exposure scenario. For example, soil photolysis will only contribute to the fate of a substance in the top few millimeters of a soil surface. Once the substance has moved below this zone to a depth that ultraviolet–visible (UV–vis) (290–800 nm) light does not penetrate, soil photolysis is not an important loss mechanism. However, mobile substances have been shown to return to the surface of the soil (i.e., back into the zone of light penetration) with the movement of water, which would extend the duration of possible photolysis (Hand et al., 2015).

Aqueous photolysis has similar uncertainties associated with it. UV–vis light penetrates to a reasonable depth in water bodies (~25 m), but its intensity decreases with depth (Morris et al., 1995). Aqueous photolysis, therefore, can be a significant process in shallow water bodies (<3–5 m), but its relevance to deeper water bodies is less clear. Additionally, the importance of direct and indirect photolysis has to be considered. In many cases, substances that do not absorb UV–vis light directly (and therefore do not undergo direct photolysis) could still be degraded photolytically through indirect photolysis (caused by free radicals generated by the absorption of UV–vis light by photosensitizing molecules e.g., humics, in natural waters) (Wallace et al., 2010).

Generally, laboratory environmental fate tests are designed to separate out single processes as much as possible and determine the degradation rate for this process in isolation. Hence, hydrolysis and photolysis studies such as the OECD TG 111 (OECD, 2004b) and OECD TG 316 (OECD, 2008a) are conducted under sterile conditions to prevent microbial metabolism. Photolysis studies are performed both in the light and in the dark to disentangle photolytic and hydrolytic degradation under identical conditions. These hydrolysis and photolysis studies are conducted in water‐only systems; therefore, the influence of soil or sediment adsorption on their significance is unknown. Similarly, biodegradation and simulation studies are generally performed in the absence of light to prevent photolysis. Where field studies are not routinely performed, this leads to a lack of understanding of the complex interplay between the different processes.

Beyond photolysis, the exclusion of light can influence the outcome of the study in another way. Most biodegradation simulation studies are conducted in the dark to prevent growth of algal populations, which may impact the heterotrophic microbial population. However, such effects are natural and their exclusion likely contributes to the decline of microbial activity in simulation studies over their 60 to 120‐day duration (Kowalczyk et al., 2015; Southwell et al., 2020). Furthermore, there is a growing body of evidence that algae can be competent degraders of xenobiotic substances in their own right, suggesting that their exclusion could skew the understanding of persistence (Ben Chekroun et al., 2014; Semple et al., 1999; Stravs et al., 2019; Thomas & Hand, 2011, 2012).

#### Temperature

The other abiotic factor to be considered is the testing temperature. In general, the temperature dependence of chemical reactions is described by the Arrhenius relationship as follows:

$$k=Ae^{-\frac{E_{a}}{RT}}$$

where *k* is the rate constant (frequency of collisions resulting in a reaction) (s^−1^), *T* is the absolute temperature (in K), *A* is the pre‐exponential factor, a constant for each chemical reaction (s^−1^), *E_a_* is the activation energy for the reaction (in the same units as RT), and *R* is the universal gas constant (J K^−1^ mol^−1^).

The Arrhenius relationship is most relevant to purely chemical reactions, such as hydrolysis (Brown, Camenzuli, et al., 2020; OECD, 2006). In that case, it can be easily addressed by conducting hydrolysis studies at three temperatures and determining a substance‐specific Arrhenius relationship. This will allow extrapolation of the degradation rate constant to any relevant environmental temperature. Photolysis, however, is generally considered to be relatively insensitive to temperature in comparison to its sensitivity to light intensity; therefore, the testing temperature for these studies is less of a concern (Ruzo et al., 1995).

In contrast, the relationship between biodegradation rates and temperature is more complex since the Arrhenius relationship does not necessarily apply to biological processes (Brown, Camenzuli, et al., 2020; Peleg et al., 2012). However, it is generally assumed that biodegradation rates are reduced at lower temperatures (represented by a Q_10_ factor, which describes the change in the degradation rate over a 10 °C temperature range). In 2007, the European Food Safety Authority (EFSA) proposed a Q_10_ conversion factor of 2.58 for conversion of biodegradation rates from studies conducted at 20 °C to 10 °C, based on the Arrhenius relationship and a median activation energy (from a range of pesticides) of 65.4 kJ mol^−1^ (EFSA, 2007). There is evidence that the type of transformation impacts the temperature dependence thereby challenging those EFSA Q_10_ conversion factors and median activation energy values (Meynet et al., 2020). Also, at least for short‐term temperature shifts, the temperature range in which the expected Arrhenius‐type behavior was observed was rather limited (Meynet et al., 2020). Currently, it is not known how temperature dependence changes if a microbial community is given sufficient time to acclimate to a given temperature.

Recently, the temperature dependence of biodegradation of hydrocarbons was assessed in detail to critically evaluate the role of temperature in the degradation of this class of substances that is ubiquitous in the environment (Brown, Camenzuli, et al., 2020). The ability of microbes to biodegrade hydrocarbons is found in extreme and temperate environments, including arctic, temperate seawater, and freshwater locations (Lewis & Prince, 2018). The data collected by Brown, Camenzuli, et al. (2020) showed that temperature dependence was still observed, but appeared to be lower than predicted by the Q_10_ factor proposed by EFSA, although it should be noted that the data compared in this review were taken from a number of studies conducted at different times. When comparing studies with systems that had been acclimated to a given temperature for a long time to studies with systems that had experienced short‐term temperature manipulations, the latter showed a stronger temperature dependence, which was close to consistent with the EFSA Q_10_ factor (Bagi et al., 2013; Ribicic et al., 2018). A potential explanation for this discrepancy could be the presence of temperature‐specific competent degrader communities in the ambient samples, which would first need to develop in the temperature‐manipulated systems, although this would need to be examined further in a study with parallel incubations at different temperatures.

### Linking simulation test outcomes to field and monitoring data

As the foregoing has demonstrated, the outcomes of laboratory tests in persistence assessment are constrained by the design and conditions imposed by such tests. Such tests are often far removed from the complex interactions present under real environmental conditions, leading to uncertainty and persistence estimates that are influenced by the test systems. This is further compounded by the difficulty to link multiple degradation processes and environmental compartments in laboratory studies. For instance, laboratory and modeling estimates of biodegradation are commonly found to be more conservative than what is actually found during monitoring studies. McDonough et al. (2018) illustrated this problem in a study evaluating the fate of amine oxide (AO), a commonly used detergent surfactant that is disposed of down the drain. The researchers conducted OECD TG 314A Sewer Water Die‐Away laboratory studies to generate primary and ultimate biodegradation rates in the sewer and OECD TG 303A Wastewater Treatment Plant Simulation Studies to quantify AO parent and metabolite levels in effluents under steady‐state conditions during wastewater treatment. The data from the laboratory studies were used in a probabilistic down‐the‐drain exposure model to estimate wastewater treatment plant effluent levels in the United States. When compared with the outcomes of an extensive monitoring campaign across the continental US in 44 WWTP effluents, results showed that AO effluent levels predicted based on laboratory studies and the best available models overestimated the measured effluent concentration by fivefold. Similarly, Honti et al. (2018), on comparing biotransformation rate constants derived from OECD TG 308 studies to those observed in the Rhine catchment (and derived from measured mass fluxes through inverse modeling), found that the laboratory‐derived rate constants were at least an order of magnitude lower than those observed in the field. The limited diversity in microbial communities present in small‐scale laboratory studies compared to field environments was, in both cases, discussed as a possible reason for the laboratory studies being more conservative than the results observed in the field. However, Kern et al. (2010) found agreement within a factor of two when comparing measured mass flows of four parent compounds and their three major transformation products (TPs) at a municipal WWTP with model‐predicted secondary effluent mass flows, which were calculated using biotransformation rate constants derived from laboratory batch experiments with activated sludge. Hence, more studies comparing laboratory to field outcomes will be needed to more thoroughly evaluate the validity of laboratory simulation studies to predict field behavior.

## CURRENT AND FUTURE OPTIONS IN PERSISTENCE ASSESSMENT

The following section describes some of the scientific advances and innovations that could be used in persistence assessment to address many of the challenges discussed in the above sections. Some of those advances that have demonstrated applicability to persistence assessments and may be useful in overcoming specific limitations or challenges have been collated and summarized in Table 3 to supplement the text in the following sections.

### Improved characterization and definition of microbial sources for biodegradation tests

#### Biomass quantification

The OECD biodegradation tests are highly prescribed and standardized in many ways, but least so with respect to the microbial biomass—the catalysts of the system. Tests designed to measure and compare substance transformation rates use biomass concentrations that can vary by orders of magnitude (see *Relevance of microbial source, sampling and sample treatment with respect to environmental conditions in the context of reducing test variability*). The OECD test guidelines stipulate that biomass is measured in one of two ways, both of which are >70 years old; one is inaccurate (plate counting [Baird et al., 2017]) and only estimates 0.01%–1% of the total cell counts, WHO, 2003) and the other is imprecise (gravimetric solids analysis—either total solids or volatile solids [Baird et al., 2017; Brown et al., 2019]). Other researchers or laboratories measure chemical oxygen demand (COD) (frequently used in wastewater treatment models, e.g., Tchobanoglous et al., 2004), organic carbon (DOC, particulate organic matter [POC] or TOC) (Brillet et al., 2018; Honti et al., 2018) or use fumigation‐induced respirometry methods (Oren et al., 2018). There are accurate and precise newer methods routinely being used in microbiology (Table S3), which get closer to measuring the catalytic element of biodegradation in the microbial biomass, enabling either standardization or normalization of the biomass used in biodegradation testing or providing further information with which to interpret their outcomes. Such methods include quantitative real‐time polymerase chain reaction targeting universal marker genes (Harms et al., 2003) and total cell counts using epifluorescence microscopy (EFM) or flow cytometry (FCM). Total cell counts using fluorescent staining of DNA with EFM are traditionally considered the “gold standard” for quantifying bacterial cells in the aqueous environment (Brown et al., 2019).

Recently, EFM and FCM were used to standardize and/or measure biomass, showing that simply increasing the number of microbial cells used in biodegradation screening tests to environmentally representative levels reduces test variability (Martin, Snape, et al., 2017; Ott, Martin, Acharya, et al., 2020; Seller et al., 2020). These studies showed that cell concentrations in standard tests vary more. Standard tests also have a lower probability of correctly assigning reference benchmark substances to their respective biodegradation class than tests using environmentally relevant inocula concentrations from: (i) activated sludge (EFM, Goodhead et al., 2014; Martin, Goodhead, et al., 2017; Martin, Snape, et al., 2017) or (ii) tests that specifically use increased cell concentrations, e.g., from seawater (EFM, Martin et al., 2018; Ott, Martin, Snape, et al., 2020; FCM, Ott, Martin, Snape, et al., 2020) to better represent the number and diversity of bacteria that a substance is likely to encounter in the environment (as mixing in surface seawaters is high, with a turnover of 10^5^–10^6^ m^3^/s and velocities of 0.05–1 m/s). The total number of microbial cells can be increased by using a higher concentration of cells or increasing the test volume, but under current EU guidance, only the latter is allowed as a so‐called enhanced biodegradation screening test, since the former is considered to lead to too favorable kinetics (R7b and R11; ECHA, 2017b, 2017a). However, when the experimental mineralization data were analyzed with respect to five biodegradation kinetic models (zero order, first order, logarithmic, logistic, and Monod no growth), the best‐fitting model that described the data well (Monod no growth) was the same for both standard RBTs and those with increased cell numbers, implying that the kinetics were not perceptibly altered (Martin, Snape, et al., 2017). Flow cytometry has a higher throughput, speed, and greater precision compared to EFM, which uses similar fluorescent DNA stains, and can distinguish live from dead cells with appropriate staining (Brown et al., 2019; Table 3). It is particularly suited to pelagic aqueous samples, where it is a standard replacement for heterotrophic plate counts in drinking water quality assessment (Van Nevel et al., 2017). Recently, an FCM method for the quantification of bacterial cells from activated sludge was developed (Brown et al., 2019).

It has been suggested that the use of biomass‐corrected pseudo‐first‐order biotransformation rates, *k*_bio_ (Honti et al., 2016) or *v*_max_ */K*_s_ (maximum specific [enzyme] growth rate/substrate saturation constant) (Trapp et al., 2018) (see *Modelling*), supports comparability between outcomes of simulation studies, and enables transfer of study outcomes to differing exposure scenarios (e.g., from a 3:1 water‐to‐sediment ratio in a standard OECD 308 sediment simulation study to a much larger water‐to‐sediment ratio in a river) (Honti et al., 2018). Such models could likely be further improved if more accurate cell counts were used instead of organic carbon or total solid measurements.

In the future, it may be possible to normalize specific biotransformation rates to the abundance and expression of the transcripts of specific enzyme groups catalyzing rate‐limiting steps, although this requires a priori knowledge of those steps and the enzymes involved, which could be obtained through statistical analyses of large enough sets of kinetic and molecular biology data from various microbial inocula (Achermann et al., 2020).

#### Biomass composition and diversity

The ability to study microbial composition and diversity was revolutionized with the discovery of universal phylogenetic markers, the ribosomal RNAs (rRNA), in the late 1970s (Woese & Fox, 1977), a roadmap for their use in the 1980s (Pace et al., 1986), and, in the last decade, by massive parallel sequencing technologies. Importantly, these techniques can analyze the presence and abundance of taxa—of the overall microbial community (e.g., 16S rRNA gene amplicons), or of the active population (e.g., 16 rRNA gene transcript amplicons)—and of specific genes encoding for catabolic enzymes (metagenomics) or their expressed transcripts (metatranscriptomics). Application of these techniques has so far demonstrated that standard OECD guideline inocula preparations reduce bacterial diversity and therefore increase the variability of screening tests (Forney et al., 2001; Goodhead et al., 2014). Conversely, an increase in the cell concentrations by filtration methods led to an increase in bacterial diversity without biasing community structure and improved the probability of correctly classifying substances based on their known biodegradation behavior in screening tests (Martin et al., 2018). Sequencing‐based methods have been used to study the relationships between microbial community metrics (Tables 3 and S3) and substance biotransformation. For instance, both taxonomic and functional diversities, in activated sludge sourced from 10 different WWTPs, were shown to correlate positively and monotonically with the average rate of primary biotransformation across 10 structurally diverse pesticides and pharmaceuticals (Johnson et al., 2015). In a follow‐up study, using more substances, a positive relationship between primary biotransformation and taxonomic and functional richness was still observed over a gradient of solids retention times (Mansfeldt et al., 2019). However, the relationship with functional diversity is perhaps more complex (Mansfeldt et al., 2019; Pholchan et al., 2013).

While biotransformation rate constants mostly correlate with diversity, such relationships can be confounded by other factors. For instance, taxonomic diversity was found to be negatively correlated with ammonia levels, biodegradable carbon {quantified as BOD_5_ [biochemical oxygen demand (milligrams of oxygen consumed per liter during five days of incubation at 20 °C)]} (Johnson et al., 2015), resource complexity, and microbial immigration (Pholchan et al., 2013), suggesting that relationships between taxonomic and functional diversity in the context of substance biotransformations are not straightforward. These results are consistent with findings from studies with soil columns simulating managed aquifer recharge, where moderately degradable substances showed increased biotransformation with increasingly refractory carbon sources (Alidina et al., 2014; D. Li et al., 2014). These trends were again aligned with more diverse communities.

It remains unclear whether increased diversity causally explains increased biotransformation rate constants. Indeed, some of these studies also report increased relative abundances of various monooxygenase‐related genes or gene transcripts (Achermann et al., 2020; Helbling et al., 2012; D. Li et al., 2014), suggesting that this, rather than general biodiversity, might be responsible for improved substance biotransformation. Therefore, attempts have been made to directly correlate different taxa (Helbling et al., 2015; Johnson et al., 2015; Wolff et al., 2018) or gene transcripts with rates of biotransformation of specific substance reactions to identify rate‐limiting taxa or enzymes, respectively (Achermann et al., 2020; Mansfeldt et al., 2019). Stable‐isotope probing using labeled substances can also be used to identify specific taxa involved in biodegradation and/or their putative transcripts (Kowalczyk et al., 2015).

Recent research has thus demonstrated the value of microbial community and diversity analyses in understanding biodegradation outcomes and biotransformation rates. Such analyses could help improve the understanding, context, and certainty of biodegradation half‐lives derived from regulatory biodegradation tests. While molecular microbial ecology techniques currently lie outside the technical remit of many industries and contract research organizations (CROs) carrying out biodegradation testing, sample storage is inexpensive and easy, sequencing costs are decreasing more quickly than Moore's law (Muir et al., 2016), and services are available that will completely process samples from nucleic acid extraction to sequence analysis (e.g., https://dnasense.com/index.php; https://microbe.med.umich.edu/microbiome-core/microbial-community-profiling; https://www.baseclear.com; https://www.northumbria.ac.uk/business-services/engage-with-us/research/nu-omics/). Data storage and interpretation are aided by the availability of publicly accessible sequence databases (e.g., National Center for Biotechnology Information [NCBI], European Molecular Biology Laboratory [EMBL]). Taking samples for such analyses (Table 3) would therefore provide resources with which to conduct future research.

Alternatively, FCM‐based community fingerprinting has recently gained attention as a more accessible and less costly alternative to sequencing‐based approaches (Barriuso et al., 2008). Such approaches use the information collected during FCM measurements to sort cells into phenotypes using different types of classification algorithms. In a recent study, Seller et al. (2021) used this approach to demonstrate the increased diversity and stability of the sediment microbial community relative to the pelagic community in OECD 308 and 309 studies, and could thus rationalize the drastically reduced interstudy variability in OECD 308 studies relative to OECD 309 studies.

Such information on community composition and diversity, be it from sequencing or FCM‐based approaches, if combined with yet uncollected metadata on environmental conditions, could help to constrain and provide more certainty on biodegradation half‐lives (see *Modelling*).

#### Future directions

In an ideal world, the half‐life of any substance in any given environment would be predictable from its structure and the microorganisms it is likely to encounter. However, the real world is more complex, and knowledge is incomplete.

##### Microbial benchmarking

In the absence of ideal predictive tools, one pragmatic approach to comparing the outcome of biodegradation tests may be to use a standardized control inoculum of known functionality and composition (OECD, 2003; Paixão et al., 2006), against which to compare reference benchmark and test chemical substances (*cf* chemical benchmarking; see *Improving validation, benchmarking and linking tests to field and monitoring data*). Such an inoculum could be composed of a consortium of known cultured bacterial members, or from a natural sample that has a complement of enzymes for a wide range of substances whose biodegradation behavior is known (e.g., activated sludge). The OECD TG 301C [MITI (I)] uses a mixed inoculum (from many different sources) that is standardized by culturing with peptone–glucose medium that unfortunately has been shown to reduce the microbial diversity in the original mixture (Forney et al., 2001). A standardized control inoculum could be made in a large batch that is lyophilized for future use by testing laboratories. One disadvantage of such an approach, aside from obtaining an inoculum with all the necessary functionalities and compositions, is the difficulty in maintaining such inocula in the long term and ensuring that they maintain the required microbial composition and functions.

##### High‐throughput biodegradation screening tests (HT‐BSTs)

Standard OECD screening tests are normally carried out in laboratory vessels (125–5000 mL; Table 1), requiring only a limited amount of replication (duplicates as a minimum), and rely on DOC or respirometry biodegradation endpoints. Such systems are time‐consuming to set up and run and thereby limit the number of substances, inocula sources, and conditions that can be screened at once. Their purpose and use as relatively simple, inexpensive, and quick “screening” tests within a tiered ITS (see Supporting Information) appear to be limited, if not flawed. Recently, a number of miniaturized HT‐BSTs have been developed akin to those becoming popular in nonanimal effects tests (ECHA, 2017c). The HT‐BSTs have been carried out using 24‐ and 96‐well plate formats with either end‐point analysis of primary (parent substance) degradation based on a colorimetric method (Martin, Goodhead, et al., 2017) or oxygen consumption (theoretical oxygen demand) using optical sensor dots (Cregut et al., 2014). The advantages of such tests are that they:

- Allow up to tens of thousands of tests in the same time as a typical RBT and therefore have the ability to research the effect of multiple microbiological and environmental factors on biodegradation (Brillet et al., 2016; Martin, Goodhead, et al., 2017).
- Are inexpensive and amenable to automation using robotic platforms (Martin, Goodhead, et al., 2017).
- Allow high replication to determine useful measures of the variability and probability of biodegradation for given substances under different conditions (Martin et al., 2018; Thouand et al., 2011).
- Allow the use of increased concentrations of inocula in 10‐fold dilutions, thereby allowing culturable, most probable number estimates of specific degraders to be quantified (Martin, Goodhead, et al., 2017; Thouand et al., 1995).

In addition to the usual disadvantages of RBTs (e.g., limited diversity—which can be counteracted by using higher inocula concentrations), HT‐BSTs have not so far been used to demonstrate mineralization and may require further validation. Such tests show potential as replacements for current RBTs.

HT‐BSTs have:

- Demonstrated how standard OECD inocula preparations with relatively low cell concentrations reduce diversity and increase test variability (Goodhead et al., 2014; Thouand et al., 1995).
- Evaluated how different inoculum sources, temperatures, and structural chemical moieties influence the biodegradation and prioritization of substances with known biodegradability (Brillet et al., 2016; Martin, Goodhead, et al., 2017; Martin et al., 2018).

An alternative approach may be to use smaller batch tests over a shorter incubation duration (i.e., 3–4 days) to obtain pseudo‐first‐order biotransformation rates that can be converted into degradation half‐lives (Fenner et al., 2020). Such systems are akin to miniature simulation tests and have the additional advantage of allowing working with mixtures of low concentrated chemicals, thus allowing the generation of consistent biotransformation data for multiple substances (see *Testing persistence at environmentally relevant low substance concentrations*) in a short period of time. The latter, however, is only possible if combined with appropriate analytical techniques, that is, high‐resolution mass spectrometry, which are still beyond the technical expertise and budget of many current CROs.

##### Identifying catalysts of chemical substance transformations

There are 100 000 chemical substances (Wang et al., 2020) and the microbial world is equally, if not more, diverse, making the identification or even prediction of enzymatic transformations challenging (Wackett & Robinson, 2020). However, the number of different functional groups that might undergo enzymatic transformation is actually more limited, and for many of these functional groups, reaction rules have been developed to predict plausible biodegradation pathways for new substances (e.g., Eawag‐BBD (Biodegradation/Biocatalysis Database) (Gao et al., 2009)). Also, these biotransformation rules have been linked to genes and enzymes potentially catalyzing these reactions (Hadadi et al., 2019; Schmid & Fenner, 2021). Knowledge of the enzyme catalysts and kinetics for a given chemical substance is likely to further improve, given this and other advances in computing, modeling, robotics, chemical analysis, and microbial bioinformatics (e.g., genomics, transcriptomics, and proteomics [Wackett & Robinson, 2020]; see also *Modelling* [Achermann et al., 2020; Zimmermann et al., 2019]).

##### The use of ecological theories

The above research demonstrates that there are empirical patterns and relationships between microbial metrics (quantity and diversity) and the biodegradability or rate of biodegradation of different substances and/or their reaction types. However, the distribution and dynamics of microorganisms containing such enzymes in any given environment are likely to be more difficult to predict, given the complexity of the microbial world. Microbial ecological theory is therefore required to transcend such situation‐bound observations and provide predictive insights (Prosser et al., 2007). Our understanding of the enzyme catalysts of substances (or the microorganisms containing them) will be improved when combined with mathematically tractable theories on their evolution, assembly, distribution, and competition.

### Overcoming hurdles with test substance

#### Passive dosing

Passive dosing technology has been used for many years to establish constant low‐level exposure concentrations for persistence testing and to mimic environmental exposure situations (Birch, Andersen, et al., 2017; Birch et al., 2018; Butler et al., 2016; Hammershøj et al., 2019, 2020; Mayer et al., 2000; Stibany et al., 2017, 2020). According to equilibrium theory, the concentration in water is proportional to the concentration in the passive dosing donor. For persistence assessments, the degradation can be evaluated in two general ways: by establishing initial low‐level concentrations in the exposure water and then removing the passive dosing medium, or by leaving the passive dosing media in the test system and tracking the loss of substance from the dosing medium, which requires evaluation of the release kinetics from the dosing material (Lee et al., 2014; Smith et al., 2012; Table 3). Reducing the exposure concentrations of substances that are toxic for microbial inocula by applying natural sorbents like clays can help in biodegradation tests to overcome inhibitory effects while enabling the use of nonspecific analyses (Nabeoka et al., 2020; Timmer et al., 2020). Passive dosing for persistence assessment is not yet included in guidance documents, but has been successfully applied in testing the ecotoxicity of poorly water‐soluble chemicals (OECD, 2019).

#### Bioavailability

**Figure 2:**
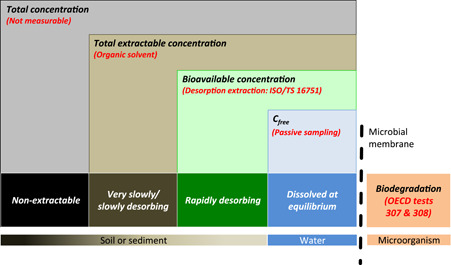
Proposal for integrating bioavailability science into OECD simulation tests, by incorporating desorption ISO methods and passive sampling determinations into the standard simulation tests for soils (OECD TG 307) and sediments (OECD TG 308). *C*_free_, freely dissolved concentration at equilibrium. Figure reproduced with permission from Ortega‐Calvo et al. (2020)

Scientific developments on bioavailability have resulted in the development of an ISO method for bioavailability measurements through desorption extraction (ISO/TS16751, 2018) and a ring‐tested protocol for determining freely dissolved concentrations in soils and sediments (Jonker et al., 2020). The integration of these approaches into standardized OECD biodegradation tests has recently been proposed (Ortega‐Calvo et al., 2020; Table 3). In this proposal (Figure 2), it is possible to assess the bioavailable fraction as a part of the total amount of substance. For example, standardized desorption extraction with Tenax (ISO/TS16751, 2018) is a robust way to determine the bioavailability and bioaccessibility of contaminants and its impact on their biodegradation in a wide set of samples from different treatments (phytoremediation, biostimulation, and bioaugmentation). With sediments (OECD TG 308), the use of passive sampling (Jonker et al., 2020) is also useful to determine bioavailable fractions, providing possibilities for connecting this knowledge with that already acquired from retrospective assessment scenarios (Burkhard & Mount, 2017). This single‐time‐point Tenax extraction ISO method can be used in, for example, the OECD TG 307 simulation test to assess persistence of bioavailable fractions of substances in soil, similarly to recent bioremediation studies (Posada‐Baquero et al., 2020).

#### Specific analysis of chemicals

Isotopic (radioactive and stable) labeled test substances and/or specific analytical methods provide the ability to dose at environmentally relevant concentrations, leading to significant advancements in understanding the persistence of chemicals beyond the use of analytical methods (O_2_ consumption, CO_2_ evolution, or DOC elimination) with significantly higher limits of quantification that do not allow for dosing at environmentally relevant concentrations (Table 3). These types of analytical methods are typically applied in cases where mono‐constituent substances are being evaluated for persistence and have failed standard screening assessments.

The value of the use of radiolabeled and stable‐labeled test substances in the investigation of NER composition and the identification of specific biodegrading microbial taxa has been discussed previously. Within the context of regulatory simulation studies, radiolabeled test substances allow for the evaluation of primary biodegradation, metabolite formation and decay, complete mineralization of the test substance, and formation of NER, as well as providing an overall mass balance to confirm the quality of the study. Information obtained from radiolabeled simulation studies can provide significant insights for persistence assessment as well as quantified rates of primary and ultimate biodegradation for use in risk assessment (Itrich & Federle, 2004; McDonough et al., 2016, 2018; Menzies et al., 2017); therefore, increased use of radiolabeled test substances would be beneficial for future persistence assessments. For example, screening studies such as ready biodegradability studies conducted with radiolabeled compounds would allow for accurate quantification of much lower levels of mineralization and demonstrate lack of persistence even for nonreadily biodegradable compounds. When connected to bioavailability research (*Bioavailability*, Figure 2), the use of radiolabeled substances provides unique ways for measuring the rates of phase exchange (e.g., slow desorption from soil or sediment). These estimations are essential for both short‐ and long‐term predictions of risk from persistent chemicals.

It is important to note that the position of the radiolabel is important in persistence assessments. In general, the radiolabel is placed in the more recalcitrant part of the molecule, but in some instances, it might be useful to place the radiolabel in other positions if there is a need to better understand the biodegradation profile of that portion of the molecule. One example is that, for molecules with multiple ring systems, separate studies might need to be performed with radiolabeling on each of the rings. This can result in very different mineralization rate estimates depending on the susceptibility of the ring to metabolism (Wang et al., 2009; Wang et al., 2013). Given this and the fact that the potential for significant mineralization over a short study duration (weeks to months) can be limited, such mineralization rates would need to be placed into the context of a more holistic view of persistence as part of a weight of evidence approach (Redman et al., 2021).

Specific analytical methods can be combined with biodegradation prediction models and known pathway information to follow metabolite formation and decay in simulation and field studies. Recent developments in the application of high‐resolution mass spectrometry in suspect and nontarget screening offer increasing possibilities to identify and quantify metabolites in such studies (Beckers et al., 2020; Brack et al., 2019; Gulde et al., 2016; Schymanski et al., 2014). This approach can be facilitated by application of isotopically labeled test compounds (both radiolabeled or stable isotopes) in studies to differentiate between the applied chemical and any potential background contamination.

#### Characterization of NERs

As explained in *Nonextractable residues (NER)* above, the thoroughly extracted soil can be further analyzed to characterize the binding mode and, if feasible, the identity of the NERs (Table 3). For this, the matrix is derivatized with reagents to disaggregate the humic matter. A silylation agent like trimethylchlorosilane is a suitable reagent for derivatization. Silylation is the introduction of a substituted silyl group (R_3_Si^−^) to molecules carrying functional groups with exchangeable protons and will lead to disaggregation of humic matter. In this way, the NER can be fractionated. Entrapped, sequestered residues (type 1 NER) will be released after the derivatization method, and covalently bound residues (type 2 NER) will remain in the matrix. However, it must be considered that the released fraction may also contain NER of a biogenic nature (type 3 NER). Recently, silylation has been applied to characterize the NERs of bisphenol S (BPS), a substitute for bisphenol A. Bisphenol S forms high amounts of NER (45% of the applied amount), of which half (51%) has been shown to be type 1 and another third (32%) type 2. Chemical analysis of the silylation extract representing type 1 NER revealed that it contains mainly the parent substance (Cao et al., 2020). In contrast, up to 15.5% of total 32% NER, formed by the herbicide pendimethalin, could be released by silylation from exhaustively extracted soil, and only trace amounts (<0.4% of applied) were related to the parent substance (Luks et al., 2021). Loeffler et al. (2020) compared the silylation and the EDTA extraction methods to release entrapped NER (type 1); 2%–12% (silylation) and 5%–18% (EDTA) of triclosan, fenoxycarb, and acetaminophen were released from soil previously extracted using PLE, and no parent substance or metabolites were detected. The authors stated that it cannot be excluded that EDTA extraction and silylation release different substances or fractions due to different release mechanisms, requiring further research on both extraction methods (i.e., silylation, EDTA). A disadvantage of the EDTA method is the extraction of high amounts of biogenic residues like proteins, phospholipids, and nucleic acids, which constitute biogenic NER (Miller & Ressler, 2005; Ogunseitan, 1993; Plassart et al., 2012; Tien et al., 1999). Therefore, no clear distinction of the three types of NER is possible when EDTA extraction is included.

Biogenic NER (BioNER) (type 3) can be quantified by hydrolyzing the matrix containing only NER under acidic conditions and elevated temperatures. Subsequently, anabolically formed amino acids carrying the radioactive ^14^C‐label can be extracted, purified, and quantified (Poßberg et al., 2016). For example, Loeffler et al. (2020) investigated NER of triclosan, fenoxycarb, and acetaminophen and released 13–36% of applied radioactivity from exhaustively extracted soil by hydrochloric acid (HCl). The hydrolysate most likely contained biogenic compounds, concluded by the authors by nondetection of the parent substances and metabolites.

The amount of formed bioNER can also be calculated from CO_2_‐release and microbial yield by applying the Microbial Turnover to Biomass (MTB) model (Trapp et al., 2018) (see *Inverse modeling to obtain biotransformation rate constants*).

One uncertainty of the NER investigation method is the choice of the extraction procedure for removing the extractable residues to obtain the matrix containing only NER. Also, an analytical procedure to identify bioNER in type 1 and type 2 fractions is so far lacking. Efforts to standardize the silylation method and the bioNER identification are ongoing. As a further weakness, the identification of parent substance and metabolites in the extracts is not easy, in part because of co‐extracted matrix components, although examples for successful identification of NER components have been reported.

#### Testing of complex substances—Carbon balance approach

Measurement of the quantity of initial organic carbon mineralized and assimilated into biomass fractions is a possible solution for the quantification of ultimate biodegradation of soluble substances and chemical mixtures, but there are limitations to the approach. By considering the remaining (nonmineralized or nonassimilated) fraction at the end of a test, biodegradability can in some cases be assessed. Drawbacks to the carbon mass balance approach include detection limits of carbon measurements and, for poorly soluble materials, discerning differences between biomass growth and test material remaining at study completion if the test material cannot be separated from the biomass. In general, the carbon balance approach has not been exploited, but is discussed in ISO 14852 Annex C as a procedure to further evaluate the complete mineralization of plastics (ISO 14852, 2018). In addition, Brillet et al. (2018) proposed a new measurement entity for evaluating the biodegradability of chemical mixtures termed Ultimately Transformed Organic Carbon (UTOC), which includes quantification of the inorganic carbon from respiration and carbon assimilated into biomass.

### Integrating abiotic transformation processes

#### Hydrolysis and photolysis

The role of hydrolysis is likely to be significant to only a relatively small number of chemicals, due to its pH dependence, and is adequately addressed by current guidance. However, the potential for photolysis should be given more prominence in the REACH weight of evidence assessment. Photolysis studies can be useful additional studies to provide a more complete understanding of potential persistence, particularly for substances that are not readily degraded by microbial metabolism. Furthermore, indirect photolysis studies have demonstrated significant degradation of substances that do not degrade through direct photolysis, and therefore, they represent a real opportunity to gain a more rounded understanding of the potential for photodegradation (Wallace et al., 2010; Table 3).

Most regulatory studies are conducted such that abiotic and biotic factors are tested separately. One exception to this philosophy is the case of the irradiated water‐sediment study (e.g., conducted according to OECD TG 308), which is an optional higher‐tier study in the EU pesticide data requirements (Commission Regulation [EU], 2013; Table 3). Degradation can be faster in this study than either the aqueous photolysis or nonirradiated OECD TG 308 study, presumably due to the impact of photosensitizers in the surface sediment and suspended solids (Katagi, 2016; Shibata et al., 2011). As such, these studies represent a powerful tool in understanding the interplay between photolysis and microbial degradation.

The surface water mineralization study according to OECD TG 309 allows the application of diffuse light to the test systems. To improve the significance of the study, by capturing the metabolic competence of any phototrophic organisms in the test water, the application of diffuse light and the use of small amounts of sediment may allow obtaining a more holistic view on the fate of a test substance in open water systems (Hand & Moreland, 2014; Hand & Oliver, 2010).

#### Temperature

Temperature‐related testing is context dependent. Simply changing the temperature in the laboratory may mean that the particular soil or sediment used in the study is exposed to a temperature to which it is not normally exposed to under field conditions and is, therefore, not adequately acclimated. As such, the key consideration should be to ensure that sample storage and the testing temperature used are appropriate to the ambient conditions from which the soils/sediments are sourced and how this affects potential changes in microbial communities, for example, soils/sediments typically exposed to colder conditions may be poorly adapted to temperatures of 20 °C and vice versa.

Additional work should reevaluate the dependence of temperature from multimedia perspective, and at different scales (lab, local, regional; also see below).

### Improving validation, benchmarking, and linking tests to field and monitoring data

While field and monitoring data provide real‐world data, sometimes, it is difficult to link those outcomes to outcomes from laboratory tests. It has been proposed that reference chemical substances with known environmental degradation behavior could be used to validate or benchmark the accuracy of laboratory tests in identifying persistent substances and avoid some of the variations in tests mentioned in the foregoing sections (e.g., Comber & Holt, 2010). Some of the set of 19 reference chemical substances with a range of properties and biodegradation behaviors proposed by Comber and Holt (2010) were successfully used to validate new biodegradation screening tests with increased microbial cell numbers (Martin, Snape, et al., 2017; Ott, Martin, Acharya, et al., 2020), demonstrating their improved accuracy and reliability. McLachlan and colleagues further extended this idea, suggesting that all laboratory tests could include a reference benchmark chemical substance against which the relative biodegradation (extent or rate) of test chemical substances could be measured (McLachlan et al., 2017), a concept that they termed benchmarking (Table 3). They further demonstrated how the concept could be used to separate degradation half‐lives from dissipation processes in the field (a lake system; see Redman et al., 2021) and outlined its use more generally in chemical hazard and risk assessments, in particular, in calibrating and translating laboratory to field data. More recently, the same group demonstrated that comparable half‐life determinations between field‐derived and OECD TG 309 simulation tests were obtained if the tests were not spiked with a given test substance, but where biodegradation of substances in the natural waters was followed by targeted (Li & McLachlan, 2019) or nontargeted chemical analyses (Li & McLachlan, 2020). Such an approach is made possible by advances in analytical chemistry (see *Specific analysis of chemicals*), but does not lend itself to new chemical substances yet to be released into the environment or those present at levels below quantification. Furthermore, to be effective and credible, benchmark substances would have similar properties and biodegradation mechanisms as those substances being tested, much in the same way that surrogate, or isotopically labeled, standards are used for validation of analytical chemistry methods.

### Modeling

Chemical regulations allow, to varying degrees, the use of models to predict fate properties, such as biotransformation. We therefore summarize recent approaches and progress in developing *predictive models that support persistence assessment* in the sense that they provide a *prediction of microbial biotransformation half‐lives and/or formation of products (e.g., TPs, NER, etc.) in different environmental compartments* as relevant in a regulatory context (i.e., agricultural soils, aquatic sediments, surface water, groundwater aquifer, etc.; Table 3). Since such models need sound experimental data to be trained and validated, we also cover recent efforts to compile *databases of microbial biotransformation* of substances.

#### Compilation of high‐quality biotransformation data

Large and curated collections of biotransformation data (i.e., information on biotransformation kinetics and pathways) are essential to further improve prediction tools. Since biotransformation rates are not intrinsic substance properties but depend on environmental or operational conditions, collections of biotransformation data should include reporting of metadata on physico‐chemical conditions (i.e., redox conditions, nutrient status, pH, temperature, organic carbon content, mineral composition, etc.) and ideally also include a characterization of biomass concentration, composition, and relevant activities. Additionally, pathway data, that is, data on the biotransformation reactions taking place, if available, are considered highly useful information because this will eventually help link kinetic information to biotransformation reactions and the putative catalyzing enzymes. Doing so requires database formats that allow storage of chemical reaction information (e.g., SMILES [Simplified Molecular Input Line Entry Specification] and SMIRKS, representing molecules and reactions, respectively [Daylight, 2020]), and information on the type and certainty of the analytical evidence supporting the reported reaction (see, e.g., Schymanski et al., 2014, for reporting of identification confidence based on LC‐MS data).

Several such data compilations, with more or less complete annotation of metadata, seem to reside with individuals or organizations (e.g., US EPA [Environmental Protection Agency] [Boethling et al., 1994], Nolte [Nolte et al., 2018], or LMC [Laboratory of Mathematical Chemistry] Oasis [Dimitrov et al., 2011; Karabunarliev et al., 2012]). However, so far, we are only aware of a couple of efforts to make these types of data compilations publicly available that is, Eawag‐BBD/PPS, formerly UM‐BBD/PPS (Eawag [formerly: University of Minnesota] Biodegradation/Biocatalysis Database and Pathway Prediction System) (Gao et al., 2009), and envipath.org (Latino et al., 2017; Wicker et al., 2016). We believe that the lack of high‐quality curated databases is related to the fact that, unlike in other related research areas, for example, in molecular biology, there is no requirement by journals in the field to deposit biotransformation data into public repositories. This leads to, first, a lack of commonly agreed formats for reporting biotransformation and related metadata, and second, difficulties in identifying funding bodies that support the maintenance and curation of biotransformation data.

#### Inverse modeling to obtain biotransformation rate constants

Assessment documents submitted in the context of different chemical regulations, some of which are publicly available (i.e., fully for pesticides, partially for industrial chemicals and pharmaceuticals), contain information from degradation tests (typically OECD TG 307, 308, or 309 simulation studies) and, therefore, in principle, represent a potentially large resource of rather consistently generated biotransformation data. Typically, required half‐life endpoints (transformation and dissipation) in one or several compartments are reported for such studies. However, it is not trivial to distill biotransformation rate constants from observed dissipation half‐lives because these may be affected by simultaneously occurring fast and slow partitioning processes that transport substances between sub‐compartments, and hence change their availability over time.

Therefore, inverse modeling approaches that explicitly account for all process kinetics (partitioning, adsorption, desorption, and transformation) have been developed (Table 3). They allow extraction of biotransformation rate constants from an in‐depth interpretation of measured data and enable the comparison of test results (Honti et al., 2016; Matthies et al., 2008). Honti et al. (2016) extracted second‐order (biomass‐corrected) biotransformation rate constants from degradation data in water‐sediment systems, using a unified model able to simulate both OECD TG 308 (sediment) and 309 (surface water) degradation test systems. In doing so, the organic carbon content was used as a proxy for biomass. A more complex approach was chosen in the “unified model for sorption and biodegradation” (Brock et al., 2019; Kästner et al., 2014), where the biomass of the degrader population is a state variable of the model. This model was applied so far only to studies where the degrader biomass has been measured as an additional variable (Brock et al., 2019; Trapp et al., 2018). In both approaches, the transformation rate constants are obtained by backward fit, but often, the calibrated parameters remain rather uncertain or even undeterminable (Honti & Fenner, 2015). Pre‐estimation of certain parameters can improve parameter determination (Brock et al., 2019).

Another issue that needs to be addressed when estimating biotransformation rate constants through inverse modeling is the formation of NER (Kästner et al., 2014) (see sections on NER). A proposed method to estimate biogenic NER (label incorporated into biomass, without any hazard potential) is the Microbial Turnover to Biomass (MTB) method (Brock et al., 2017; Schäffer et al., 2018; Trapp et al., 2018). When a ^14^C‐labeled substrate is mineralized, the label will either remain in the biomass (yield Y) or in CO_2_ (fraction 1 − Y). Theoretical yields can be calculated from thermodynamics (Gibbs energy) and structural data. Together with the measured CO_2_ evolution in degradation tests and data on the microbial biomass, biogenic NER formation can be estimated. Further work with the model is required that would demonstrate and improve understanding of the influence of intrinsic properties of the substance and the environmental matrix on NER formation. A validation exercise, demonstrating the suitability of the model and improving confidence in the data generated, is ongoing.

#### Advances and novel approaches in QSBR development

Generally, the field of quantitative–structure–biodegradation relationship (QSBR) development has matured from mostly using multivariate, linear modeling approaches to using more sophisticated machine‐learning approaches (Di Guardo et al., 2018; Mamy et al., 2015). However, any development of QSBRs suffers from small and nonhomogeneous databases and widely varying rate constants. Therefore, recent efforts in the QSBR development have sought smart strategies to overcome these data limitations. Three major directions have been attempted: (i) joining of data sets across different study conditions and even environmental compartments, although this requires normalization of data to account for differences in physico‐chemical conditions and biomass concentration and composition, which is a major challenge as discussed below; (ii) inclusion of prior knowledge to group substances into more homogeneous groups to obtain significant relationships within such groups; and (iii) grouping of substances according to their structural properties for prediction of physico‐chemical properties and environmental fate endpoints (Acharya et al., 2019; Mansouri et al., 2019).

Normalizing biotransformation data sets to join them requires knowing what the major influencing factors are and how they quantitatively affect the observed biotransformation rate constants. Correcting for biomass concentration using second‐order transformation rate constants yields (ideally, and if the microbial communities are similar) more universally valid kinetic information than first‐order rate constants (Honti et al., 2016). They might even be, to some extent, compartment‐independent (Shrestha et al., 2016) and hence are the preferred input for multicompartment model systems. In fact, kinetic parameters for microbial degradation of phenanthrene obtained by inverse modeling were rather similar for experiments with four different degrader strains, despite varying initial biomass (Adam et al., 2014, Rein et al., 2016). Measures of total biomass can be used to derive second‐order rate constants, but do not allow consideration of differences in biomass composition and/or relevant activities. Therefore, recent attempts have focused on more specifically quantifying the degrader population (e.g., by quantifying incorporation of a labeled substance into amino acids or phospholipid fatty acids [Nowak et al., 2013]), or to identify and quantify the enzymes catalyzing the observed biotransformation reaction (e.g., Achermann et al., 2020; Zimmermann et al., 2019). However, for most degradation studies, characterization of degrader biomass is not provided at all.

Other influencing factors that have been normalized for are temperature (EFSA, 2007) and bioavailable concentrations (Honti et al., 2016; Shrestha et al., 2016). Yet, although work is ongoing into temperature‐adapted and temperature‐manipulated systems for assessment of hydrocarbon substances (Brown, Camenzuli, et al., 2020), as of now, there is a lack of sufficient experimental data to derive more refined approaches for temperature correction. The bioavailable concentration that drives microbial degradation is often approximated as the freely dissolved substance concentration (i.e., calculated from sorption equilibrium considerations using experimentally determined or estimated sorption coefficients). More sophisticated and nuanced discussions on defining bioavailable fractions of substances and proposed frameworks of definition can be found in Kickham et al., 2012; Ortega‐Calvo et al., 2015, 2020 (see *Bioavailability* section). Strongly adsorbing substances, including hydrophobic but also some charged substances, are known to slowly desorb, which can limit the degradation process (Adam et al., 2014; Rein et al., 2016; Wick et al., 2001). Therefore, the “Unified Model for Sorption and Biodegradation” (Brock et al., 2019; Kästner et al., 2014; Trapp et al., 2018) has two adsorption compartments with slow and fast ad/desorption. The actual bioavailable concentration may affect observed biotransformation rate constants in at least two ways: too high concentrations may inhibit the microbial community, due to toxic effects (Hammershøj et al., 2019, 2020), while too low substance concentrations and/or that are not bioavailable can lead to slow metabolism and eventually starve the degrader population (Kundu et al., 2019; Rein et al., 2016; Toräng et al., 2003). Finally, Nolte et al. (2020) proposed a way to correct biotransformation rate constants for concentration‐dependent adaptation. However, we believe that this approach should be validated further and become more mechanistically underpinned before being applied.

Since quantitatively correcting for all influencing factors when joining data sets for improved model development will remain challenging, benchmarking, that is, the consideration of relative rather than absolute biotransformation behavior, has been suggested as an alternative approach to join biotransformation data from different experimental assays, without the need for explicit data normalization (see *Improving validation, benchmarking and linking tests to field and monitoring data*). However, it was also noted that relative behavior can only be expected to be constant across systems for groups of substances subject to the same influencing factors. Thus, to apply this approach confidently, it would again require a certain level of mechanistic understanding, similarly as for the normalization approach. This said, as sufficiently large data sets are being accumulated for different environmental compartments (i.e., agricultural soil [Latino et al., 2017], activated sludge [envipath.org]), the validity of constant relative behavior across conditions and environmental compartments should certainly be further scrutinized. Recently, it was found that the average half‐lives for 40 diverse plant protection products in agricultural soils can be reasonably well predicted from their half‐lives measured in activated sludge from two wastewater treatment plants, if corrected for estimated differences in bioavailability (Fenner et al., 2020).

An alternative to creating larger data sets is to structure the available data based on first principles as far as possible, with the hope of deriving more significant QSBRs for more homogeneous subgroups of substances. For biotransformation, one obvious approach would be to subdivide the substances into groups of substances known or hypothesized to undergo the same or similar transformation reactions. This is supported by recent evidence that chemicals undergoing the same type of enzymatic transformation indeed show similar relative changes in biotransformation kinetics across different activated sludge communities (Achermann et al., 2018). Nolte et al. (2018) and Wang et al. (2018) have recently used the Eawag‐BBD/PPS system (Gao et al., 2011) to cluster substances based on predicted biotransformation reactions. They showed that they could develop more significant multivariate‐type QSBRs within those subgroups as compared to QSBRs developed with all compounds in the data set.

Overall, new experimental methods that allow for high‐throughput experimental determination of consistent degradation information for mixtures of substances (e.g., Achermann et al., 2018; Birch et al., 2018) will support further exploration of factors influencing biotransformation and lead to improved prediction algorithms for degradation half‐times and pathways.

## TRANSLATING SCIENCE INTO REGULATION

Environmental regulation of chemicals relies on laboratory studies performed according to internationally accepted guidelines such as those published by OECD, ISO, ASTM (American Society for Testing and Materials), OPPTS (US Office of Prevention, Pesticides and Toxic Substances), or JMAFF (Japanese Ministry of Agriculture, Forestry, and Fisheries). Guideline studies are considered to provide standardized and comparable results for the majority of substances undergoing safety assessment and generate results that can be directly compared against internationally recognized regulatory criteria. These studies are customarily performed by specialized private CROs to a GLP standard under controlled conditions. Subsequently, reports and data are mutually acceptable to different global regulatory bodies. These studies usually obtain high Klimisch scores of reliability (Klimisch et al., 1997). Since they have become required as part of regulatory data packages for all substance regimes, these studies have grown in number, as has the experience of the laboratories performing them.

### Ratification of new methods

Translating the science into regulation requires the recognition of new methods and guidance in interpretation by the international regulatory community. The guidelines for regulatory test methods for chemical hazard assessments typically require ratification by an internationally recognized organization (e.g., OECD, ISO, OPPTS, and others) prior to their acceptance. For the OECD, only Member Countries, the European Commission, or the Secretariat can submit new test guideline proposals (Rasmussen et al., 2019). If accepted, the proposal goes through a process of validation and review to demonstrate proof of concept and reliability of the proposed method (OECD, 1995). This requires strong stakeholder engagement, extensive funding, and time commitment throughout. New proposals are discussed annually and, even if no further validation studies are required, the test guideline adoption procedure takes at least two years. The full development of test guidelines often exceeds 10 years encompassing identification of requirement, evaluation and proof of concept, inter‐laboratory validation, final ring‐testing, acceptance, and publication (e.g., OECD TG 306 Nyholm & Kristensen, 1987; OECD, 1992b). Consequently, the science contained in a “new” test guideline may be over a decade old. This partially explains the previously described discrepancy between available methods and existing test guidelines.

Ratification of new methods is a crucial way to develop accurate and reliable tests. However, it should be acknowledged that (i) not all OECD tests were developed and validated using substances of known behavior that went on to be reported in open‐access texts (e.g., the OECD TG 307, 308, and 309), (ii) ratification by the OECD does not equate to adoption in all regulatory frameworks e.g., data generated from an OECD TG 314 (OECD, 2008b) cannot generate a definitive conclusion for a REACH persistence assessment, but can be accepted as a weight‐of‐evidence approach (ECHA, 2017a) (it does, however, generate data that are very useful for exposure assessments); and (iii) not all proposed method updates require the same depth of review; for instance, ECETOC has previously proposed for an OECD expert working group to consolidate and update RBTs to reflect availability of new instrumentation with increased analytical sensitivity (ECETOC, 2013b). Where techniques have been reviewed and validated for use in other sectors, inclusion in existing OECD test guidelines could be fast‐tracked. However, this would depend on the evidence provided to prove suitability for inclusion and buy‐in from OECD member nations.

Any updates of existing tests, replacement tests, or alternative strategies for assessment must offer sufficient (precautionary) environmental protection as existing assessments. Additional guidance would need to explain how data could be consistently interpreted and used in regulatory assessments.

### Developing and using new methods and techniques

Until test guidelines for persistence assessment are ratified or updated, scientists can use established but nonstandardized techniques (see *Current and future options in persistence assessment*) to provide more insight into a substance's persistence, which regulators evaluate as weight‐of‐evidence data. As a consequence of the time required prior to publication of the guidance and adoption into regulatory guidance, there will be variance between methodology and analytical techniques adopted by academia and/or industry and those recommended in test guidelines. Techniques may not be known or adopted by CROs because there has been no demand; specialized skills and equipment may be required; and the cost of investment in this is weighted against requirement. While many techniques become less expensive and more accessible (e.g., DNA sequencing; see *Biomass composition and diversity*), mechanisms are missing to upskill and provide new knowledge to CROs and regulators for use in persistence assessments.

In contrast, academic researchers often possess the necessary technical expertise but may not publish sufficient meta‐ and/or raw data to allow regulators to use their studies for assessment (Moermond et al., 2016; Wang et al., 2018; also see *Compilation of high‐quality biotransformation data*). Where academic work is to be published that could be used to support regulatory decisions (e.g., degradation of a single substance in a specific compartment), we would encourage that researchers and their funding bodies recognize the OECD reporting requirements. Dialogue between the OECD and journal publishers should be encouraged to provide support in writing guidance, to authors and potentially stipulate it as part of the publisher's terms and conditions. Similar approaches are being implemented for reporting of statistics and metagenomic studies (Eckert et al., 2020; Veldkamp et al., 2014). That said, the research community is beginning to understand the necessity and advantages of validity criteria (e.g., Klimisch scoring) and robust reporting that allow studies to be evaluated as key supporting evidence in persistence assessments or as part of a weight of evidence. Such improved understanding between regulators, academics, and researchers of the requirements of data quality and comparability will drive forward research in this area. Specifically, reporting of biotransformation study outcomes should ideally be in line with required endpoints that have to be reported under REACH, that is, (1) primary degradation rate, (2) degradation half‐lives (t_1/2_), (3) disappearance or dissipation half‐lives (DT50), (4) route and rate of transformation for substance and associated TPs, and (5) NERs.

Where standard testing is not the best option (e.g., difficult‐to‐test substances), discussions between applicants and authorities on tailor‐made nonstandard study designs should be considered, applying the concept of “reviewing and accepting study plans before results are known” developed by Chambers (2019) to academic research in general. Prior agreement on standard operating procedures and describing planned analysis for degradation studies before conducting the experiment could help to (i) improve study design, (ii) ensure publication of null or negative results, (iii) avoid cherry‐picking of results, and finally, (iv) engender regulatory trust in the data generated.

Scientists should aim to engage regulators at an early stage of method development and validation to receive feedback and better adapt methods toward improving regulatory frameworks and assessments. Regulators should be encouraged to actively engage in scientific progress. Guidance documents (e.g., ECHA, 2017b) should be updated in a timely fashion to reflect improved understanding. Academia, industry, and regulatory bodies need to collaborate more effectively to exchange their knowledge to progress and improve testing methods toward more predictive and robust assessments.

## CONCLUSIONS

In recent years, the field of biodegradation science has made significant advances that could help to improve the precision and accuracy of persistence assessments. However, without effectively transforming the advances into standard test methods that receive regulatory acceptance and guidance and/or use of the knowledge to better inform assessments, their value is limited. This can be achieved by academia, regulatory bodies, and industry working together more efficiently so it no longer takes >10 years for new science to be incorporated into methods and achieve regulatory acceptance sometime later. Science can help to develop robust, technically acceptable methods so that the appropriate decisions can be made regarding the persistence of test substances. Persistence is a key environmental attribute used in evaluating the fate and risks of chemicals in the environment, but it is nontrivial and complex, therefore deserving application of the best available science in a timely and robust manner.

## CONFLICT OF INTEREST

The authors declare that there are no conflicts of interest.

## Supporting information

This article contains online‐only Supporting Information.

 The Supporting Information provides information on the evolution of persistence assessments in European regulatory frameworks and the current state of knowledge on persistence assessment within that context. It aims to inform knowledgeable but less expert members of the scientific and regulatory community on the context of the critical review, which refers to this Supporting Information.

## Data Availability

This manuscript is a critical review and does not contain any new or newly analyzed data, but instead refers to data published elsewhere.
