# Supplementary material for: Scientific concepts and methods for moving persistence assessments into the 21st century
Source: Integr Environ Assess Manag. 2022 Feb 23;18(6):1454–87. doi: 10.1002/ieam.4575 (PMC9790601; doi:10.1002/ieam.4575)
Supplement: Supplementary file 1 — The Supporting Information provides information on the evolution of persistence assessments in European regulatory frameworks and the current state of knowledge on persistence assessment within that context. It aims to inform knowledgeable but less expert members of the scientific and regulatory community on the context of the critical review, which refers to this Supporting Information. [file IEAM-18-1454-s001.docx]

**Scientific concepts and methods for moving persistence assessments into the 21^st^ Century**

# Evolution of persistence assessment in European Regulatory Frameworks

Under European Regulatory Frameworks, the approach taken for assessing persistence has evolved over many years (Figure S1). With regards to industrial and consumer substances, initially disparate legislations evolved into the REACH Regulation (EC No. 1907/2006, Annex XIII) which set out definitions for assessing persistence based on half-life values in different environmental compartments.

Prior to REACH, two pieces of legislation regulated the manufacture and supply of industrial and consumer substances in the EU: the Notification of New Substances Regulations (NONS, an amendment of the Dangerous Substances Directive; 67/548/EEC), which required producers and importers to submit notifications to individual European Member States, and the Existing Substances Regulation (ESR, (EEC) No. 793/93), which set up a review process for priority substances.

Risk assessment for new substances and prioritized existing substances was performed by regulators using a technical guidance document that was updated in 2003 to include a marine scenario and related PBT assessment requirement (European Commission Joint Research Centre, 2003). The PBT (persistent, bioaccumulative, toxic) criteria were adapted and modified from the framework developed under OSPAR, with vPvB (very persistent, very bioaccumulative) criteria added. An interim strategy for the management of PBT and vPvB substances was agreed by European Member States in 2004, which set up a PBT sub-group of the Technical Committee for New & Existing Substances (TCNES), which was composed of both regulators and industry, to assess, test and identify such substances. Member States were responsible for carrying out the PBT assessment, which relied on the expert judgement of the sub-group as no formal guidance on performing the assessment existed. Existing substances in the IUCLID database (International Uniform Chemical Information Database) were screened manually by the Member States, and a PBT assessment was also performed for substances still undergoing review under the ESR priority lists in addition to screening and identifying all new substances requiring further PBT assessment. All new substances had to have a base-set of data by the time they were supplied at > 1 tonnes/year including biodegradation, acute ecotoxicity and bioaccumulation (log *K_ow_*) (ECHA, 2008).

When the REACH Regulation came into force in the EU (The European Parliament and the Council of the European Union, 2006) it included legal criteria for identification of PBT and vPvB substances in Annex XIII, which was amended in 2011 to permit a weight of evidence to be used in the PBT assessment.

Under REACH, chemical industry registrants are responsible for assessing the safety of their substances, which includes assessing PBT and vPvB properties. This is a major difference from the preceding legislation. ECHA’s R11 guidance (ECHA, 2017a), which has been revised multiple times since its original publication, details how this evaluation should be performed.

A number of assessments could not be finalized before the New and Existing Substance legislation was superseded by REACH. Some of which were completed by Member States during the transition period, with most of the remaining substances being added to the Community Rolling Action Plan (CoRAP) to allow Member States to review the PBT assessment performed by the registrants. A few have been evaluated by Member States on a voluntary basis outside of the CoRAP process (e.g. selected siloxanes and Dechlorane plus).

Prioritization of substances for Substance Evaluation under REACH was set out from the beginning in terms of registered supply tonnage in conjunction with exposure potential, *in silico* screening, and annual manual screening of substances identified as possible candidates from the registration database for PBT and vPvB properties. This is performed at Member State Committee level with input from the Expert Groups. Substances that flag as potential PBT/vPvBs can be placed on the CoRAP to be distributed amongst Member States for formal evaluation amongst others that are selected.


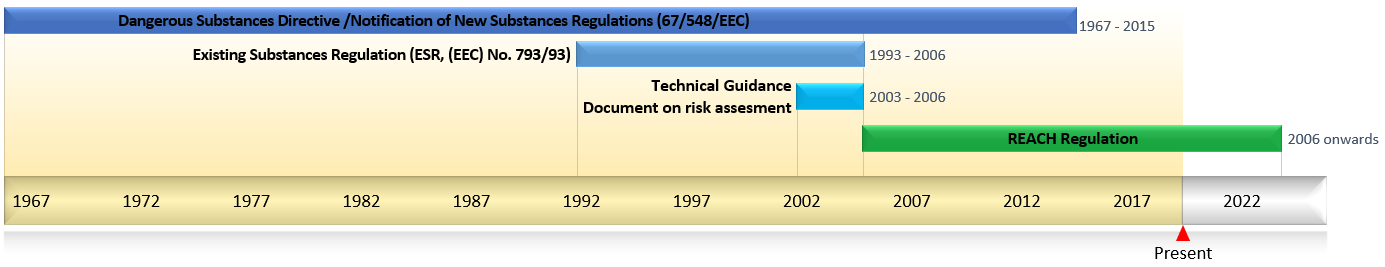
Figure S1: Chronology of EU legislation linked to persistence assessments.

# Current state of knowledge

## Persistence assessment - screening

It is important to emphasize that all relevant data from a REACH registrant must be examined to derive a conclusion of “not P/ not vP” or “may fulfil the P or vP criteria” or “P/vP” across all environmental compartments of soil, water and sediment where data permits (Figure S2). Screening data and supporting evidence as defined in REACH guidance text R11, R7a and R7b (ECHA, 2017a, 2017b, 2017c) tabulated below (Table S1) can only be used to conclude that a substance is not environmentally persistent (See Table 1 in main text for more details).

Table S1: Screening data and accepted supporting evidence utilised in persistence assessment (adapted from Table. 11-4, (ECHA, 2017a)).

| Appropriate Method | | Outcome | Potential limitations |
| --- | --- | --- | --- |
| Evidence of Ready Biodegradability  Activated Sludge /WWTP effluent/river water/soil inoculum | OECD TG 301 A to F/ OECD TG 310 (OECD TG 306 for marine water) | Pass indicates the substance is not persistent | Many as discussed in the paper (e.g. microbial diversity, relevance to complex substances, poorly water-soluble substances etc). |
| Evidence of Biodegradability (Enhanced screening tests*)  Prolongation of study duration, increased volume of test media etc.  Activated Sludge/ WWTP effluent/ /river water/soil/sea water inoculum | OECD TG 301 A to F / OECD TG 310 / OECD TG 306 | Pass indicates the substance is not persistent |  |
| Inherent Biodegradability  Activated Sludge/ WWTP effluent/ /river water/soil/sea water/sediment inoculum | OECD TG 302 B & C | Pass indicates the substance may not be persistent (specific ECHA criteria) | As per evidence of ready biodegradability.  After a lag phase (should be no longer than 3 days), the level of 70% mineralization must be reached within 7 days or 14 days. These methods are not adapted for substances with a limited bioavailability such as poorly water-soluble substances. |
| Other non-experimental data (WoE) | EPI suite™ models BIOWIN™, EAWAG-BBD pathway prediction system, OECD QSAR Toolbox etc.  Read-across from analogue substance(s) – common transformation products | Can be used to further consider persistence potential | Issues with regulatory acceptance, standard approaches, not suitable for all substances, increased costs if not accepted etc. |
| Laboratory derived weight-of-evidence (WoE) data | Log K_oc_ /hydrolysis/photolysis etc.  Pure culture data  Other degradation studies e.g. anaerobic, sewage treatment simulation  Abiotic degradation at environmentally relevant pH and temperature (Hydrolysis, Photodegradation, Redox)  Field-scale monitoring/biomonitoring |  | As per other non-experimental data (WoE) |

* During the preparation of the 2017 updates of R7b. (version 4), increasing the biomass concentration and inoculum density was excluded from the study design for enhanced ready biodegradability studies. The increased ratio of substance to inoculum was deemed to be too favorable by the European Chemical Agency (R7b.) This exclusion has been seen controversially as research has shown that 100-fold increased cell concentration was not too favorable (Ott et al., 2020b, 2020a).

## Persistence assessment - definitive

Definitive criteria, (REACH Annex XIII) can be employed to derive a conclusion of “P/vP” or “not P/vP” in that respective compartment. Definitive data ideally takes the form of laboratory simulation test data (Table S2). Under REACH, simulation tests (water and/or sediment and/or soil; see Table 2 in main text for more details) are often required depending on the substance’s intrinsic properties, exposure conditions and tonnage band (unless a suitable reason for waiving them is available e.g. if readily biodegradable). ECHA and/or Member States have the power to request such studies where necessary.

Table S2: Definitive data utilized in persistence assessment (adapted from Table. 11-5, (ECHA, 2017a)).

| Appropriate Method | | Endpoints | Potential limitations |
| --- | --- | --- | --- |
| Simulation test: Aerobic and anaerobic transformation in soil | OECD TG 307 | CO_2_ ↑  Primary half-life (t½; days)  Non-extractable residues (NER)  Transformation products | Issues with substance volatility, NER formation, radio-labelling etc. |
| Simulation test: Aerobic and anaerobic transformation in aquatic sediment systems | OECD TG 308 | CO_2_ ↑  Primary half-life (t½; days)  NER  Transformation products |  |
| Simulation test: Aerobic mineralisation surface water – simulation biodegradation test | OECD TG 309 | CO_2_ ↑  Primary half-life (t½; days)  NER in suspended sediment test |  |

## Persistence assessment – performance and conclusions

All available data are examined to derive a conclusion for “not P/ not vP” or “may fulfil the P or vP criteria” or “P/vP” across all environmental compartments of soil, water and sediment. Conclusions of persistence for a substance have further implications for a REACH registrant i.e. requirements for further studies such as assessment of bioaccumulation potential and future vertebrate studies and increasing evaluative work that takes both extensive periods of time and quantities of money. If designated as PBT/vPvB, this can eventually lead to a loss of market access as a worst case. As such it is very important that false positive conclusions of persistence (i.e. considering a substance as potentially persistent when it is not) are avoided.

In comparison to the persistence assessment or PBT/vPvB assessment under plant protection product and medicinal regulations, REACH is generally data poor in terms of higher-tier simulation tests, as such tests usually are only required by higher tonnage band registrants and only after a formal testing proposal is submitted to ECHA.

## Persistence assessment - data interpretation and evidence

Under REACH, if an initial ready biodegradation screen is not passed, persistence conclusions often rely heavily on a weight-of-evidence determination utilizing experience, precedent and expert judgement (Figure S2). This should enable assessment of all available information with and without direct numerical values to be compared with the criteria set out in Annex XIII (The European Parliament and the Council of the European Union, 2006). There are numerous publications and guidance documents available (e.g. Brandt et al., 2016; ECETOC, 2014; ECHA, 2020; OECD, 2019). However, formal weight-of-evidence methods and approaches are not fully available and existing ones could be improved upon to assist companies.

The weight of evidence approach relies on there being sufficient evidence to conclude on any hazard endpoint (Redman et al., 2021). Additional scrutiny is required to assess reliability, relevance and the robustness of data that has been provided for any persistence conclusion. One example is data produced by quantitative structure–activity relationships (QSARs), which do not have adequate training sets for classes of substances being assessed. Data generated for UVCBs and substances with unusual chemistries have to be scrutinized carefully if using QSARs and may be scrutinized by regulators. As part of a WoE a registrant may also provide additional information from varying sources e.g. published literature, read-across from substance analogues, data from existing studies, *in vitro* studies, epidemiological data/human experience etc., which may allow regulators to conclude without the need for additional data. This is especially important for difficult to test substances that will require significant amendment to design for OECD studies.


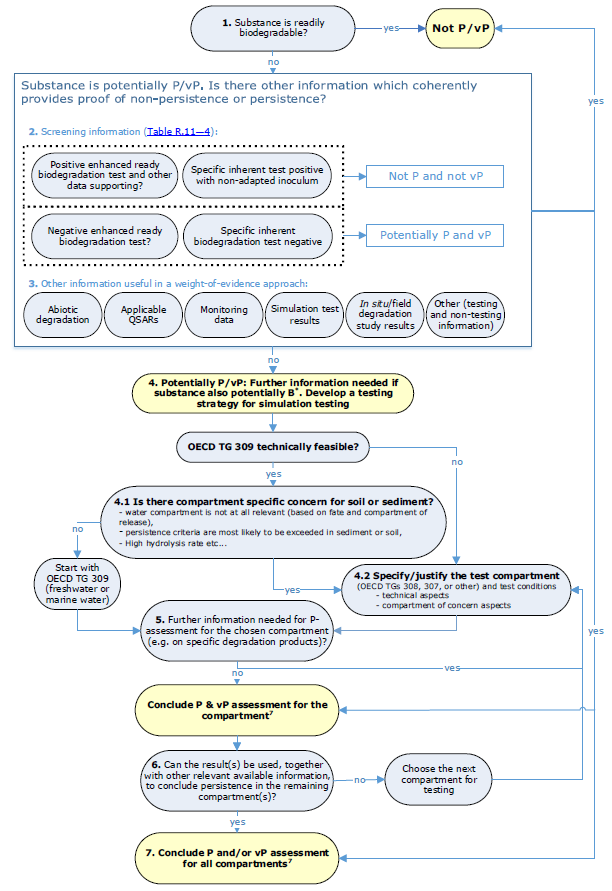


Figure S2: Integrated Testing Strategy for persistence assessment – maximizing data use and targeting testing (Figure R.11-3, (ECHA, 2017a)).

Table S3. Metrics and techniques used in other fields of study for the characterization of natural microbial samples (Lyon et al., 2020).

| **Inoculum Criterion** | **Metric** | **Possible Method (Metric)** | **Comment** |
| --- | --- | --- | --- |
| **Microbial Load** | Colony forming units (CFU/mL) | Basic microbiology techniques, like Dip Slides or plating (Colony Forming Unit/mL) or Most Probable Number (MPN) | Relatively cheap and simple process. Will not capture the majority of microbes which are non-culturable |
|  | Cells/mL | Flow cytometry (cells/mL); optical density or fluorescence | Requires flow cytometer. Will not easily differentiate viable vs dead cells |
| **Biodiversity** | Biodiversity indices (Shannon, Simpson, H, etc.) | DNA sequencing data (many options available) | Cost varies based on technique (Kowalczyk et al., 2015) |
|  |  | Functional Gene Array (GeoChip 5.0) | Covers ~1500 functional gene families. Specialized laboratory needed |
| **Metabolic Activity** | Biodegradation of a positive control | Inclusion of biodegradable analogues to the test substance | Indication of presence of competent degraders |
|  | Soil indices based on enzyme activity and respiration in soil | Enzyme activity number (EAN), Metabolic quotient, Basal respiration (qCO2 – respiration to microbial biomass ratio), etc. | see Vázquez-Rodríguez *et al.* (2007) |
|  | Substrate metabolism profile | Biolog plates – contain multiple carbon sources | Easy to read colorimetric assay |

# References

Brandt, M., Becker, E., Jöhncke, U., Sättler, D., Schulte, C., 2016. A weight-of-evidence approach to assess chemicals: case study on the assessment of persistence of 4,6-substituted phenolic benzotriazoles in the environment. Environ. Sci. Eur. 28, 1–14. https://doi.org/10.1186/s12302-016-0072-y

ECETOC, 2014. Information to be considered in a weight-of-evidence-based PBT/vPvB assessment of chemicals (Annex XIII of REACH). ECETOC, Brussels.

ECHA, 2020. Weight of evidence [WWW Document]. URL https://echa.europa.eu/support/registration/how-to-avoid-unnecessary-testing-on-animals/weight-of-evidence (accessed 5.8.20).

ECHA, 2017a. Guidance on information requirements and chemical safety assessment, chapter R.11: PBT/vPvB assessment (version 3.0). European Chemical Agency, Helsinki, Finland. https://doi.org/10.2823/128621

ECHA, 2017b. Guidance on information requirements and chemical safety assessment Chapter R . 7a : Endpoint specific guidance (version 6.0). https://doi.org/10.2823/84188

ECHA, 2017c. Guidance on information requirements and chemical safety assessment, chapter R . 7b : endpoint specific guidance (version 4.0), Echa. Helsinki. https://doi.org/10.2823/84188

ECHA, 2008. Guidance on information requirements and chemical safety assessment Part C: PBT Assessment.

European Commission Joint Research Centre, 2003. Technical guidance document on risk assessment, Part II. EUR 20418 EN/2.

Kowalczyk, A., Martin, T.J., Price, O.R., Snape, J.R., van Egmond, R.A., Finnegan, C.J., Schäfer, H., Davenport, R.J., Bending, G.D., Schafer, H., Davenport, R.J., Bending, G.D., 2015. Refinement of biodegradation tests methodologies and the proposed utility of new microbial ecology techniques. Ecotoxicol. Environ. Saf. 111, 9–22. https://doi.org/10.1016/j.ecoenv.2014.09.021

Lyon, D., Saunders, D.M., Maloney, E., Smit, M.G., 2020. Poster: Qualification of a microbial inoculum for OECD Ready Biodegradability Tests (RBTs), in: SETAC Europe 30th Annual Meeting.

OECD, 2019. Guiding Principles an Key Elements For Establishing A Weight of Evidence for Chemical Assessment No. 311. https://images.chemycal.com/Media/Files/ENV-JM-MONO(2019)31.pdf 1–37.

Ott, A., Martin, T.J., Acharya, K., Lyon, D.Y., Robinson, N., Rowles, B., Snape, J.R., Still, I., Whale, G.F., Albright, V.C., Bäverbäck, P., Best, N., Commander, R., Eickhoff, C., Finn, S., Hidding, B., Maischak, H., Sowders, K.A., Taruki, M., Walton, H.E., Wennberg, A.C., Davenport, R.J., Albright 3rd, V.C., Baverback, P., Best, N., Commander, R., Eickhoff, C., Finn, S., Hidding, B., Maischak, H., Sowders, K.A., Taruki, M., Walton, H.E., Wennberg, A.C., Davenport, R.J., Albright, V.C., Bäverbäck, P., Best, N., Commander, R., Eickhoff, C., Finn, S., Hidding, B., Maischak, H., Sowders, K.A., Taruki, M., Walton, H.E., Wennberg, A.C., Davenport, R.J., Albright-III, V.C., Bäverbäck, P., Best, N., Commander, R., Eickhoff, C., Finn, S., Hidding, B., Maischak, H., Sowders, K.A., Taruki, M., Walton, H.E., Wennberg, A.C., Davenport, R.J., 2020a. Multi-laboratory Validation of a New Marine Biodegradation Screening Test for Chemical Persistence Assessment. Environ. Sci. Technol. 54, 4210–4220. https://doi.org/10.1021/acs.est.9b07710

Ott, A., Martin, T.J., Snape, J.R., Davenport, R.J., 2020b. Increased cell numbers improve marine biodegradation tests for persistence assessment. Sci. Total Environ. 706, 135621. https://doi.org/10.1016/j.scitotenv.2019.135621

Redman, A., Bietz, J., Davis, J., Lyon, D.Y., Maloney, E., Ott, A., Otte, J., Palais, F., Parsons, J., Wang, N., 2021. Conceptual framework for moving persistence (P) assessments into the 21st Century.

The European Parliament and the Council of the European Union, 2006. REGULATION (EC) No 1907/2006 OF THE EUROPEAN PARLIAMENT AND OF THE COUNCIL of 18 December 2006 concerning the Registration, Evaluation, Authorisation and Restriction of Chemicals (REACH), Official Journal of the European Union. https://doi.org/http://eur-lex.europa.eu/LexUriServ/LexUriServ.do?uri=OJ:L:2006:396:0001:0849:EN:PDF

Vázquez-Rodríguez, G.A., Garabétian, F., Rols, J.L., 2007. Inocula from activated sludge for ready biodegradability testing: Homogenization by preconditioning. Chemosphere 68, 1447–1454. https://doi.org/10.1016/j.chemosphere.2007.03.073
